# Integration of MALDI‐TOF MS and 16S rRNA Analysis for Identification of Plant‐Based Fermentation‐Associated Microbiota

**DOI:** 10.1111/1758-2229.70237

**Published:** 2026-01-27

**Authors:** Agnieszka Ludwiczak, Ewelina Sibińska, Iwona Adamczyk, Miłosz Wasicki, Oleksandra Pryshchepa, Michał Złoch, Klaudia Grygorowicz, Małgorzata Szultka‐Młyńska, Paweł Pomastowski

**Affiliations:** ^1^ Centre for Modern Interdisciplinary Technologies Nicolaus Copernicus University in Toruń Toruń Poland; ^2^ Department of Immunology, Faculty of Biological and Veterinary Sciences Nicolaus Copernicus University in Toruń Toruń Poland; ^3^ Department of Physiology and Toxicology Kazimierz Wielki University Bydgoszcz Poland; ^4^ Department of Environmental Chemistry and Bioanalytics, Faculty of Chemistry Nicolaus Copernicus University in Toruń Toruń Poland

**Keywords:** fermentation, LAB, MALDI‐TOF MS, plant‐based products, 16S rRNA sequencing

## Abstract

The specific fermented matrices influence microbial diversity and proteomic adaptations being crucial for optimising fermentation efficiency and effective microbial identification. Therefore, the study aimed to investigate the impact of plant‐based fermentation matrices and their physicochemical composition on microbial diversity and MS‐protein profiles. Microbial communities were characterised using MALDI‐TOF MS and 16S rRNA sequencing. Physicochemical analyses were conducted on the 10 fermentation matrices. The sequencing verified low‐confidence MALDI identifications and assessed species‐level microbial diversity. Combined MALDI‐TOF MS and 16S rRNA gene sequencing confirmed the presence of 24 species across five taxonomic classes and revealed strong matrix‐dependent variation in the lactic acid bacteria composition. A significant positive correlation was observed between *Lactiplantibacillus pentosus* abundance and pH, with the presence being negatively associated with Ca and Mg levels in the fermented products. Furthermore, the concentration of carbohydrates and Fe was positively correlated with *Corynebacterium amycolatum* and *Micrococcus luteus* . MALDI−TOF MS spectra obtained for the key lactic acid bacteria species revealed differences in protein profiles depending on the type of fermented matrices. The study provides new insights into the interactions between microbial communities and fermentation substrates, emphasising the role of physicochemical properties of plant‐based matrices in shaping microbial diversity and proteomic adaptations.

## Graphical abstract

Plant‐based fermentation matrices significantly influence microbial diversity and species‐specific proteomic adaptations. The integration of MALDI‐TOF MS and 16S rRNA sequencing with physicochemical analyses allowed for a better understanding of the impact of specific fermentation products on differences in microbial diversity and spectral protein profiles of the identified bacterial species.

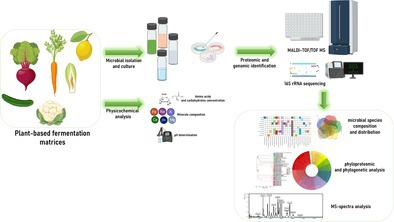

## 1 Introduction

Modern approaches to a healthy lifestyle and balanced nutrition increasingly emphasise the importance of natural and traditional methods of food preservation, placing fermented foods as an ecologically responsible choice compared to industrially produced dietary supplements (Leeuwendaal et al. 2022). In this context, fermented foods hold a special place, combining taste, health benefits, and ecological value. The fermentation process leads to the production of probiotics, vitamins, antioxidants and free amino acids. Additionally, fermented products retain or even concentrate carbohydrates and essential minerals, including potassium, sodium, iron, and magnesium. These nutrients may originate from the raw materials used in fermentation, such as vegetables and fruit, being further synthesised by the microbial activity that enhances their bioavailability (Marco et al. 2017).

The lactic acid fermentation is a fundamental biochemical pathway responsible for the preservation, sensory and health properties of fermented foods. The process involves the breakdown of sugars naturally present in vegetables and fruit into lactic acid by the action of lactic acid bacteria (LAB) (Gänzle 2015; Adebo et al. 2022). *Lactiplantibacillus plantarum*, *Lactobacillus acidophilus* , *Lacticaseibacillus casei*, and *Bifidobacterium bifidum* are among the most studied probiotics within the LAB group. Metabolic conversion driven by the LAB bacteria is responsible for the characteristic sour taste of fermented products and is a natural preservation method by lowering the pH, which inhibits the growth of spoilage microorganisms and pathogens (Rao et al. 2020; Mastuki et al. 2024). The low pH environment created by lactic acid stabilises the final product and acts synergistically with other bioactive components, such as free amino acids, carbohydrates and minerals, to deliver additional physiological benefits (Gänzle 2015). Different fermentation substrates create distinct ecological niches, shaping the composition and metabolic activity of microbial communities. Therefore, identifying how specific fermented products influence microbial succession can provide new insights into fermentation dynamics.

The adaptation of microorganisms to challenging environmental conditions during the fermentation process is crucial for their survival and metabolic activity. Microbial cells are exposed to significant osmotic stress in the fermentation due to the accumulation of salt ions and organic acids. High mineral concentrations create a hypertonic environment responsible for water loss from microbial cells (Papadimitriou et al. 2016). However, the LAB evolved mechanisms to counteract the osmotic stress, such as the accumulation of compatible solutes (e.g., proline, betaine) and the selective uptake of K^+^ to maintain the osmotic balance. The adaptations to overcome the osmotic, ionic, and acidic stress are driven by proteomic changes, including the modulation of protein profiles and specific protein signatures that enable the LAB to survive and thrive under challenging fermentation conditions (Kleerebezem et al. 2010; Mejía‐Caballero et al. 2025).

Among the available analytical protein‐based methods, Matrix‐Assisted Laser Desorption/Ionisation Time‐of‐Flight Mass Spectrometry (MALDI‐TOF MS) emerged as a powerful and efficient tool for the microbiome and proteomic analysis, offering distinct advantages over traditional and high‐throughput proteomics techniques such as LC–MS/MS (Wenning et al. 2014). MALDI‐TOF MS, as a modern technique, demonstrates high efficiency in identifying the microbial composition of products like ferments, cheeses, yoghurts, or beer, ensuring their quality and safety (Schumann and Maier 2014; Singhal et al. 2015). Unlike traditional culture‐based methods or 16S rRNA sequencing requiring extensive processing and sequencing steps, MALDI‐TOF MS enables direct bacterial identification within minutes by comparing protein mass spectra with extensive reference databases (Singhal et al. 2015). This feature is particularly valuable for fermented food microbiome studies, where microbial populations are highly diverse and dynamic. However, fermentation involves a complex community of LAB, yeasts, and other microbes, many of which belong to the same genus, but exhibit matrix‐specific metabolic adaptations leading to inconsistencies in species‐ or genus identification. Misclassification or lower identification occurs due to proteomic differences between the analysed species and the reference strains in the database (Wenning et al. 2014). Therefore, the analysis of the extent of variation in mass spectral signatures of LAB isolated from different fermented matrices can provide new insights into how fermentation environments influence protein profiles. Understanding the proteomic adaptation can help optimise fermentation conditions to enhance flavour, texture, and probiotic properties in fermented foods.

Given the significant role of the physicochemical composition of the fermentation matrices in shaping microbial communities, the aim of the study was to characterise the microbial diversity and species‐level composition of spontaneous plant fermentation using a combined approach of the proteomic‐based identification technique (MALDI‐TOF MS) and 16S rRNA gene sequencing. Matrix‐dependent proteomic profiles were also investigated to identify how the physicochemical composition of plant‐based fermented substrates influences bacterial MS‐protein profiles and influences the accuracy of microbial identification. By linking the MALDI‐derived spectral complexity of the isolated LAB with the composition of fermented plant material, the study provides novel insights into how fermentation environments shape both microbial communities and their proteomic signatures.

## 2 Materials and Methods

### 2.1 Sources of the Fermented Food

The study material included 10 different vegetable−based fermented products sourced from three Polish family−owned companies specialising in fruit and vegetable processing. The companies adhere to traditional home fermentation recipes handed down through generations. Additionally, among these fermented products, three types of cucumbers and beetroot were prepared independently according to a homemade recipe in Poland.

A total of 10 fermented plant‐based samples were investigated, including three types of fermented cucumbers along with beetroot, carrot, cauliflower, celery, kimchi, lemon and radish. Each type of fermented plant matrice was prepared in three independent batches (biological triplicates) under identical conditions to ensure the reproducibility of the fermentation process. The fermentation process was carried out without the use of preservatives, artificial colourants, sugar, or flavour enhancers. Only salt (20 g of Kłodawa rock salt, Poland per 1 L of tap water), and natural spices commonly used in fermentation were applied, including oak leaves, cherry leaves, dill, horseradish, pepper, mustard seeds, bay leaves, and allspice. The fermented products were stored for 1 month in glass jars or barrels in a saline solution in the dark prior to the analysis. The products were unpasteurised, allowing the fermentation process to remain active for a longer time. After 1 month of fermentation, the fermented products were maintained at 4°C before the analysis.

### 2.2 Microorganism Culture

Each fermented sample was divided into its liquid and solid fractions, which were analysed separately. The liquid fraction was collected through pipetting after mixing the sample, while the solid fraction was homogenised and processed separately. The isolation of microorganisms was carried out using a combination of liquid and solid media under various incubation conditions to maximise the diversity of cultivable microorganisms.

The liquid media utilised for bacteria culture included the peptone water, BSM Broth with BSM Supplement, and MRS Broth with Tween 80, selected for their suitability in cultivating diverse microbial populations. The solid media comprised MacConkey Agar, Columbia Blood Agar with the addition of 5% defibrinated sheep blood, TOS‐Propionate Agar with lithium mupiricin supplement and Bifido Selective Supplement B, Raka Ray Agar with the LAB selective supplement and the Cycloheximide solution, BSM Agar with BSM Supplement, M‐17 Agar, and MRS Agar, providing a range of nutrient compositions and selective properties to support the isolation and differentiation of various bacterial species.

One millilitre or one gram of sample was added to a tube containing 9 mL of sterile liquid medium and mixed briefly by vortexing (1 h for solid fraction). For each sample, a series of dilutions was prepared ranging from 10^−1^ to 10^−4^. Samples and all their dilutions were applied to different culture media using the grated plating method. 100 μL of the appropriate dilution was applied to the medium and evenly distributed using a spreader. For BSM Agar and MRS Agar, a 1‐day pre‐incubation at 30°C was performed in the corresponding liquid media to enhance microbial recovery. For the remaining media, samples were plated directly from the peptone water. All the cell cultures were then incubated at 30°C (18–48 h) under different atmospheric conditions. McConkey Agar was incubated under aerobic conditions, Columbia Blood Agar in a CO_2_‐enriched atmosphere (5%), and all other media under anaerobic conditions using sachet containers to generate an oxygen‐free atmosphere (AnaeroGen 2.5 L).

### 2.3 MALDI Analysis

Single colonies were selected from the cell culture based on morphological differences, and subcultures were prepared on the same media to obtain pure cultures. The identification of the isolated strains was performed using the Microflex LT MALDI–TOF/TOF mass spectrometer (Bruker Daltonik GmbH, Germany) by a direct sample deposition onto a plate using a sterile loop. The isolated colony was spread as a thin layer directly on the plate's surface. Then, 1 μL of 70% formic acid was applied to the spot, and after drying, 1 μL of the HCCA matrix solution (concentration of 10 mg/mL in a solvent containing 50% acetonitrile, 47.5% water, and 2.5% trifluoroacetic acid) was added. The obtained spectra were analysed using FlexControl software with MBT Compass version 4.1 software (Bruker Daltonics). The Bacterial Test Standard (Bruker, Germany) was used as the calibrator (Sibińska et al. 2025). The identification results were interpreted based on the score values generated by the MALDI Biotyper software, following the manufacturer's guidelines and previously published criteria (Bourassa and Butler‐Wu 2015; Sibińska et al. 2025). Specifically, the score values ≥ 2.00 were considered highly reliable for species‐level identification and were classified as the consistency category A. Scores ranging from 1.70 to 1.99 were indicative of probable genus‐level identification (consistency category B), while scores < 1.70 were considered unreliable.

### 2.4 Isolation and 16S rRNA Sequencing

The isolation of the bacterial DNA was performed using the E.Z.N.A. Bacterial DNA Kit (Omega Bio‐tek, GA, USA) according to the manufacturer's protocol with small modifications (Ludwiczak et al. 2025). Briefly, overnight‐cultivated bacteria colonies were collected from Petri dishes by sterile inoculation loops. Then, 10 μL of lysozyme (50 mg/mL) was added to the probes. After 30 min of incubation at 37°C, 2 μL of lysostaphin (15 U/μL) was added. Later, 100 μL of TL Buffer and 20 μL of Proteinase K solution were mixed (45 min at 55°C). RNA residues were digested with 5 μL of RNase A (5 min, RT). The HiBind column was used for the DNA binding, and elution was performed after heating the column at 65°C for 5 min to enhance the DNA recovery. A total of 50 μL of the preheated Elution Buffer was used for the elution. The eluted DNA was subsequently stored at −20°C.

The purity and concentration of the DNA following isolation were assessed using a NanoDrop 2000c UV–Vis spectrophotometer (Thermo Fisher Scientific). Samples with an A_260/280_ ratio between 1.8 and 2.0 were used for the PCR amplification. The primers 27F (5′‐AGA GTT TGA TCC TGG CTC AG‐3′) and 1492R (5′‐GGT TAC CTT GTT ACG ACT T‐3′) were used for amplification of the 16S rRNA gene and sequencing (Lane 1991). PCR reactions were carried out using the Taq PCR Master Mix (2×) (Qiagen), with 10 ng of DNA. The thermal cycling conditions were as follows: initial denaturation (94°C, 3 min), 30 cycles of: denaturation (94°C, 30 s), annealing (55°C, 30 s), extension (72°C, 90 s), final extension (72°C, 7 min). The analysis of the PCR product's size was conducted on a 1.0% w/v agarose gel with SYBR Green I Nucleic Acid Gel Stain using Invitrogen 100 bp DNA Ladder as a marker. Sequencing of the 16S rRNA fragments utilised the 27F and 1492R universal primers (Lane 1991). UVP Biospectrum 810 Imaging System with dedicated software was applied for the visualisation of amplificons. Illumina sequencing was performed by Genomed S.A. (Poland). The MaSuRCA assembler was used for processing and assembling DNA sequencing reads (Zimin et al. 2013). Sequence alignments were refined manually (e.g., correcting gaps or mismatches), and conserved regions were integrated for accurate identification in the FASTA−compatible format in the BioEdit software (version 7.2.5). Aligned sequences were compared against a reference database NCBI 16S rRNA/ITS database. The searching of nucleotide databases using a nucleotide query (Blastn) was performed excluding models (XM/XP) and uncultured/environmental sample sequences. It was limited to sequences from the typed material.

### 2.5 Physicochemical Analysis

#### 2.5.1 pH Measurements

The Mettler Toledo SevenCompact S220 pH meter was used for the precise measurement of pH in fermented vegetables. Prior to the analysis, the device was calibrated using the standard pH buffers (pH 4.01, 7.00 and 9.21). All the measurements were conducted at a controlled temperature of 25°C.

#### 2.5.2 Determination of Carbohydrate

The phenol−sulphuric acid method was used for quantifying total sugars (Masuko et al. 2005). The carbohydrate content was conducted for 50 μL of filtered liquid fermented material after the addition of 150 μL of sulfuric acid (96%–98%) and 30 μL of 5% phenol. The incubation was carried out in a thermal block at 90°C for 5 min. After the incubation, the samples were cooled at room temperature for 5 min. Two hundred microlitres of the sample was applied to the 96‐well microplate. The measurements were performed using a ThermoScientific VarioLux spectrophotometer with the SkanIT Re 5.0 program at a wavelength of 490 nm. The calibration curve for lactose was prepared within the concentration range of 0–350 mM. The calibration curve equation for lactose was determined as *y* = 0.0317*x* + 0.0216 with a coefficient of determination (*R* ^2^) of 0.9994.

#### 2.5.3 Determination of Amino Acids

The amino acid concentration was determined according to a previously described method (Lee and Drescher 1978). OPA reagent was freshly prepared by dissolving 3.8 g of borax in 80 mL of water, adding 2 mL of ethanol, 2 mL of β‐mercaptoethanol, 0.1 g of SDS and 0.08 g of o‐phthaldialdehyde and filling to 100 mL. Five hundred microlitres of each sample was mixed with 1 mL of 0.75 M TCA and centrifuged (14.800 × g, 10 min). Fifty microlitres of the supernatant were transferred to wells of a 96‐well microplate, mixed with 150 μL of OPA reagent and incubated at RT for 4 min. The measurements were performed at 340 nm wavelength against the OPA reagent as a blank. The standard curve for glycine was prepared within the concentration range of 0–30 mg/mL. The calibration equation was *y* = 0.508*x* + 0.104 with a coefficient of determination (*R* ^2^) of 0.9984.

#### 2.5.4 Determination of Minerals Composition

To identify the mineral profile, 50 μL of liquid and 50 mg of solid materials were added to 200 μL of 65% H_2_NO_3_. The samples were incubated for 2 h at 90°C with mixing. Subsequently, the samples were quantitatively transferred to 10 mL LC–MS grade water. The diluted samples were centrifuged at 4000 x g for 5 min, then 8 mL of the supernatant was collected without disturbing the pellet, and the concentrations of minerals: Na, K, Ca, Mg, Fe, Zn were measured (Paul et al. Paul et al. 2017). A calibration curve for each mineral was prepared using Supelco Certipur ICP multi‐element standard solution (1000 mg/L), by creating a series of dilutions in the range of 5–1000 μg/L for six elements against a 2% H_2_NO_3_ blank. ICP‐OES AVIO 220 Max was employed with the following conditions applied: 8 L/min plasma gas argon radiofrequency, power at 1500 W, nebulizer gas flow rate 0.7 L/min. The microelements were monitored at lines: Ca317.933, Fe238.204, K766.490, Mg285.213, Na589.592 and Zn206.200.

### 2.6 Statistical Analysis

Data analysis and chart generation were performed using the PS IMAGO PRO 9.0 software package (version 29.0.0.0, Predictive Solutions, Poland) integrated with IBM SPSS Statistics. Furthermore, Python (version 3.8) was utilised alongside the Pandas library (version 1.2.0) for data processing and Matplotlib (version 3.4.0) for visualisation. Each physicochemical analysis was performed in triplicate. The Kruskal−Wallis test was employed to assess the statistically significant differences in microbial diversity across the different types of fermented materials. To identify which specific groups exhibited significant differences, pairwise comparisons were conducted using Dunn's post−hoc test with the Bonferroni correction. The statistical significance of the correlation coefficients was determined using the Pearson correlation significance test with *n* − 2 degrees of freedom. Two biodiversity indices were calculated (the Shannon−Wiener Index and the Simpson Index), to assess microbial diversity and community structure in the analysed fermentation environments. The Shannon Index was used to evaluate species richness and evenness, providing insight into the distribution of species within the community (Shannon 1948). The Simpson Index was applied to measure species dominance. Nonmetric multidimensional scaling (NMDS) was applied to visualise the relationships among different types of fermented products based on their *m*/*z* profiles and intensity. The distance matrix was computed using the aggregated mean values across all data points (*m*/*z* and intensity) and the Bray–Curtis dissimilarity metric.

The phyloproteomic dendrogram was generated using the MALDI Biotyper Compass Explorer 4.1 software, using a similarity algorithm based on the analysis of MALDI‐TOF MS spectra. The construction of the dendrogram was based on the previously generated Main Spectra Profiles (MSP), which are representative spectral profiles for the studied strains. The evolutionary tree of identified bacterial strains was created based on the Neighbour‐Joining and Maximum Composite Likelihood method using MEGA7software (Kumar et al. 2016) and was visualised using Interactive Tree of Life (iTOL) v 6.5.4 (Letunic and Bork 2016).

The identification of selected proteins was performed by querying the UniProt Knowledgebase (UniProtKB) using mass‐to‐charge (*m*/*z*) values derived from MALDI‐TOF MS spectra (Bateman et al. 2025). The analysis focused on peptide signals present in isolates from at least three different plant‐based fermentation matrices. For each selected *m*/*z* signal, a database search was conducted to determine whether the signal was associated with the target bacterial species' protein.

## 3 Results

**FIGURE 1:**
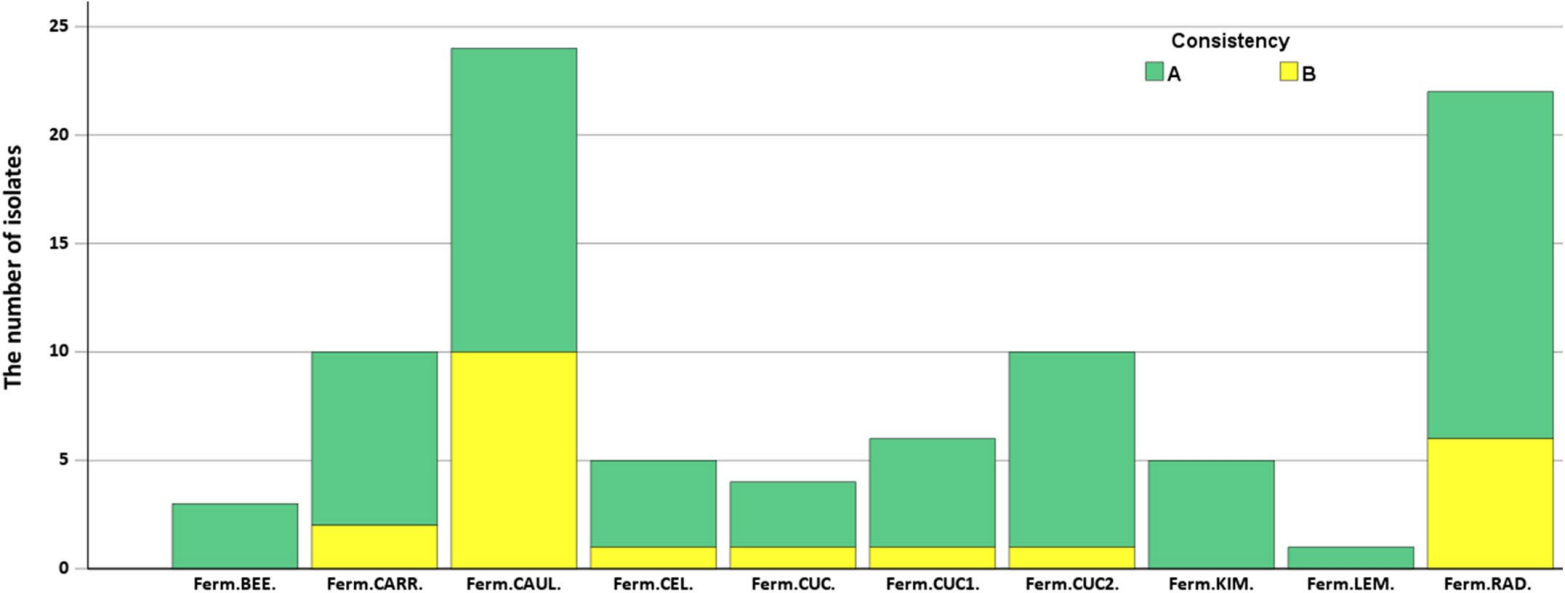
The number of isolates obtained from different fermented products, categorised according to consistency categories (A—high consistency, highly probable species identification and B—low consistency [probable genus identification]). Fermented materials: Ferm.BEE.‐ fermented beetroot, Ferm.CARR.‐ fermented carrot, Ferm.CAUL.‐ fermented cauliflower, Ferm.CEL.‐ fermented celery, Ferm.CUC.‐ fermented cucumber, Ferm.CUC1.‐ fermented cucumber1, Ferm.CUC2.‐ fermented cucumber2, Ferm.KIM.‐ fermented kimchi, Ferm.LEM.‐ fermented lemon, Ferm.RAD.‐ fermented radish.

The number of obtained isolates varied depending on the type of fermented material (Figure 1). A total of 90 isolates were obtained from all the plant‐based fermented matrices with the majority of isolates (*N* = 68) identified at the species level. Among all the samples, the highest number of isolates was recovered from the fermented cauliflower (*N* = 24), with a predominance of high‐consistency species identification (*N* = 14). Similarly, a high number of isolates was observed in the fermented radish (*N* = 22), with a higher proportion of A consistency compared to B (*N* = 16 and *N* = 6, respectively). In contrast, only a single isolate was obtained from the fermented lemon. Beetroot, cucumber, and kimchi also yielded relatively few isolates (*N* = 3, 4 and 5, respectively), with beetroot and kimchi containing only isolates classified at high consistency at the species level. Interestingly, the number of isolates obtained from fermented cucumbers varied depending on the source of the obtained products. The highest number (*N* = 10) was obtained from the cucumber2 sample.

**FIGURE 2:**
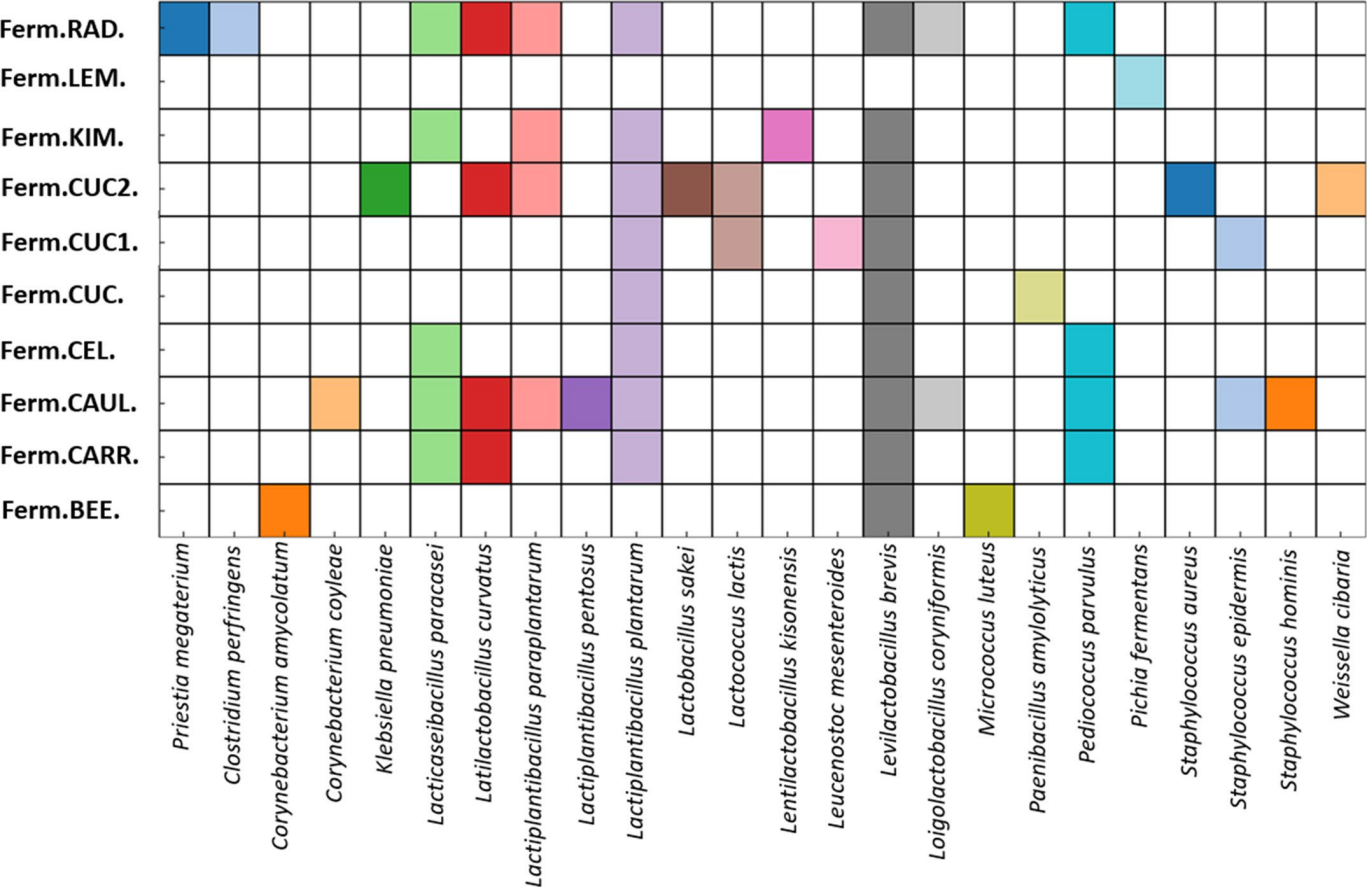
The occurrence of the species identified in plant‐based fermented products. Individual species were marked with a unique colour on the heatmap. For a detailed explanation of the abbreviations used, please see Figure 1.

The MALDI TOF‐MS analysis revealed a diversity of 24 distinct microbial species classified into five classes (Figure 2). *Bacilli* class dominated the microbial community (*N* = 84), with the remaining isolates classified to following classes: *Actinomycetia* (*N* = 2), *Clostridia* (*N* = 1), *Gammaproteobacteria* (*N* = 1) and *Saccharomycetes* (N = 2) (Figure S1). The predominantly isolated bacterial species included *Lactiplantibacillus plantarum* (12 isolates), *Lacticaseibacillus paracasei* (8 isolates), *Lactiplantibacillus paraplantarum* (4 isolates), *Pediococcus parvulus* (6 isolates) and *Latilactobacillus curvatus* (4 isolates). The growth patterns of key bacterial species on the selective media showed that *L. brevis* predominantly grew on RakaRay and MRS media, *L. paracasei* exhibited growth on BSM medium. In addition, *L. plantarum* was detected on BSM and MRS media and *L. curvatus* was selectively cultivated on TOS medium.

Cauliflower was the most abundant source of microorganisms, with 11 species identification and 5 genus identification representing the *Bacillus* genus (Figure 2). The isolates represented *Lacticaseibacillus* ( *L. paracasei* ), *Lactiplantibacillus* (*L. plantarum, L. paraplantarum, L. pentosus*) and *Levilactobacillus* ( *L. brevis* ). Fourteen isolates were identified by MALDI TOF − MS from the fermented radish. Six of these were classified at the genus level, representing *Lacticaseibacillus, Lentilactobacillus, Pediococcus* sp., *Peribacillus* sp. and *Levilactobacillus*. The most dominant species were *Lacticaseibacillus paracasei* (*N* = 3) and *Lactiplantibacillus plantarum* (*N* = 3). Among the three types of fermented cucumbers, the highest number of identified isolates was obtained from cucumber2 (*N* = 10), with only one isolate remaining unidentified at the species level. The most abundant taxa belonged to the *Latilactobacillus* and *Lactiplantibacillus* genus, including *L. curvatus, L. sakei, L. paraplantarum and L. plantarum*. The fermented lemon exhibited the lowest microbial abundance, with only a single yeast species, *Pichia fermentans* , being identified.

**FIGURE 3:**
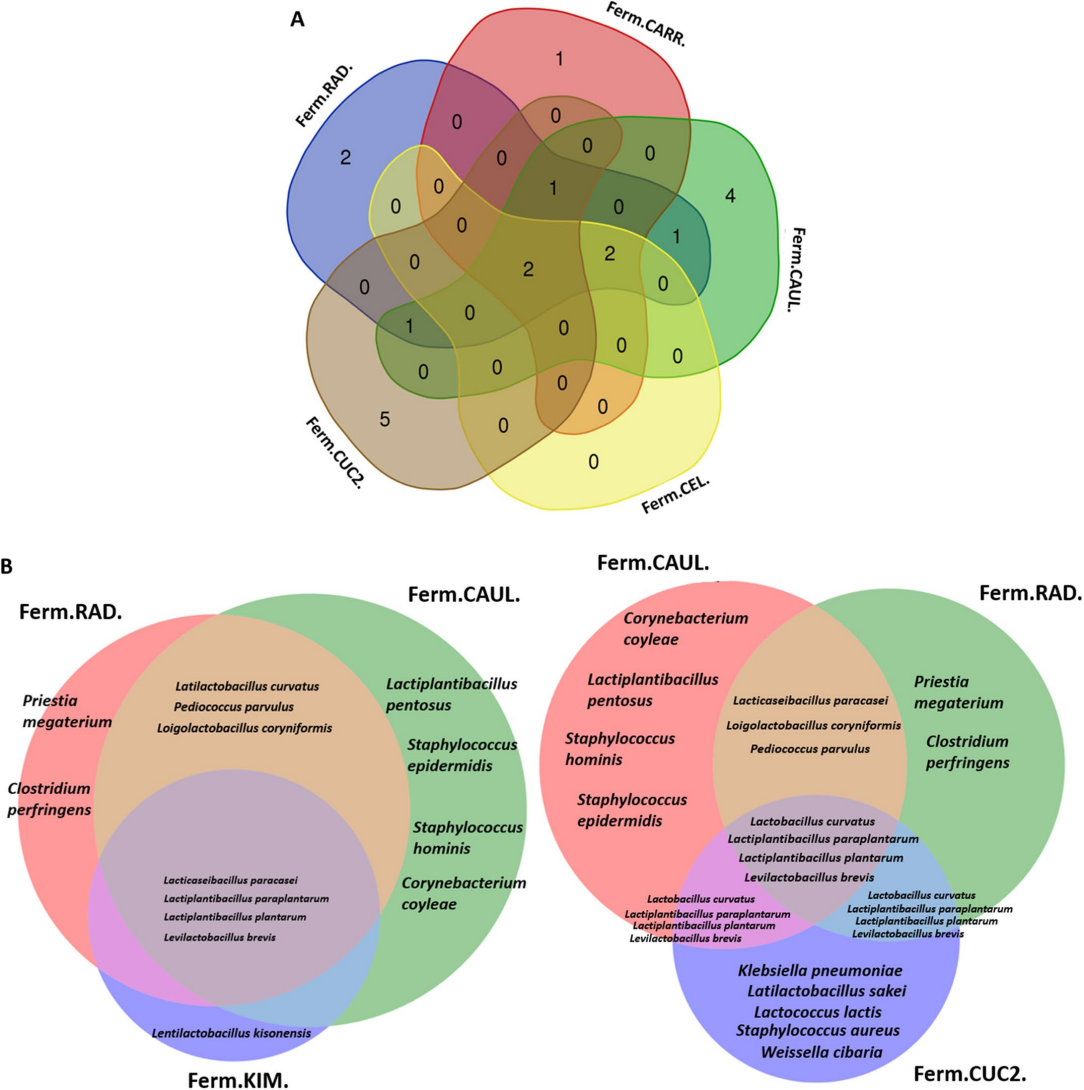
Microbial species distribution across fermented products with the highest microbial diversity was demonstrated as a Venn diagram. The number of common and unique microbial species depending on the isolation source was presented across five (A) and three (B) fermented sources. For a detailed explanation of the abbreviations used, please see Figure 1.

The most widely distributed species among the five most abundant types of plant‐based fermented products were *Lactiplantibacillus plantarum* and *Levilactobacillus brevis* (Figures 2 and 3). Some species like *Paenibacillus amylolyticus, Klebsiella pneumoniae, Leuconostoc mesenteroides*, and *Corynebacterium amycolatum* , were identified specifically in one type of the fermented product, respectively in the cucumber, cucumber2, cucumber1 and beetroot. The highest number of unique microbial species, which contributed to the differentiation of the plant‐based fermented products microbiome was identified among the cauliflower, radish and cucumber2 (Figure 3B). Four species (*C. coylae, L. pentosus, S. hominis* and *S. epidermidis* ) were identified exclusively in the cauliflower. The cucumber2 contained five unique species that were not identified in any other fermented product: *K. pneumoniae*, *L. sakei*, *L. lactis*, *S. aureus* and *W. cibaria* (Figure 3A,B). Some microorganisms were widely distributed across most fermented products. For instance, *L. plantarum* was absent only in the beetroot and lemon, while *L. brevis* was not identified only in the lemon. Among the three fermentation matrices with the highest microbial diversity (cauliflower, radish and cucumber2), four species were common to all three matrices: *L. curvatus, L. paraplantarum, L. plantarum* and *L. brevis*. Additionally, *L. paracasei, L. paraplantarum, L. plantarum*, and *L. brevis* were found in the radish, cauliflower and kimchi (Figure 3B).

The MALDI‐TOF MS results were validated by the 16S rRNA gene sequencing for isolates identified with low‐confidence scores (< 2.00) and selected isolates with high‐confidence (≥ 2.00), confirming the complementary application of both proteomic and genomic approaches for bacterial identification. The majority of MALDI‐based identifications were consistent with those obtained through the 16S rRNA gene sequencing (Table S1). For the identification scores ≥ 2.00 and consistency category A, the sequence identity of the best‐matching 16S rRNA sequences ranged from 99.52% for *Loigolactobacillus coryniformis* to 100% for *Lactiplantibacillus paraplantarum*. For two isolates identified by the MALDI system as *Pediococcus parvulus* at the genus and species levels, the sequence identity to the reference 16S rRNA gene was lower, respectively 97.6% and 98.63%. Consequently, the 16S rRNA analysis also provided reliable identification only at the genus level. In the case of certain strains, including *Lactiplantibacillus paraplantarum, Lentilactobacillus buchneri*, and *Levilactobacillus brevis*, despite the MALDI identifications yielding a score below 2.00 and being classified as consistency category B, the species assignments suggested by the MALDI system were congruent with those obtained through the 16S rRNA sequencing. For some isolates of *Levilactobacillus brevis and Pediococcus ethanolidurans* identified at the genus level with consistency B, MALDI identifications at the genus level were confirmed, and the 16S rRNA sequencing allowed for species‐level identification. Conversely, the MALDI identification of one isolate as *Pediococcus parvulus* was incorrect, even at the genus level. The 16S rRNA sequencing enabled the accurate identification of the isolate as *Latilactobacillus curvatus*.

**FIGURE 4:**
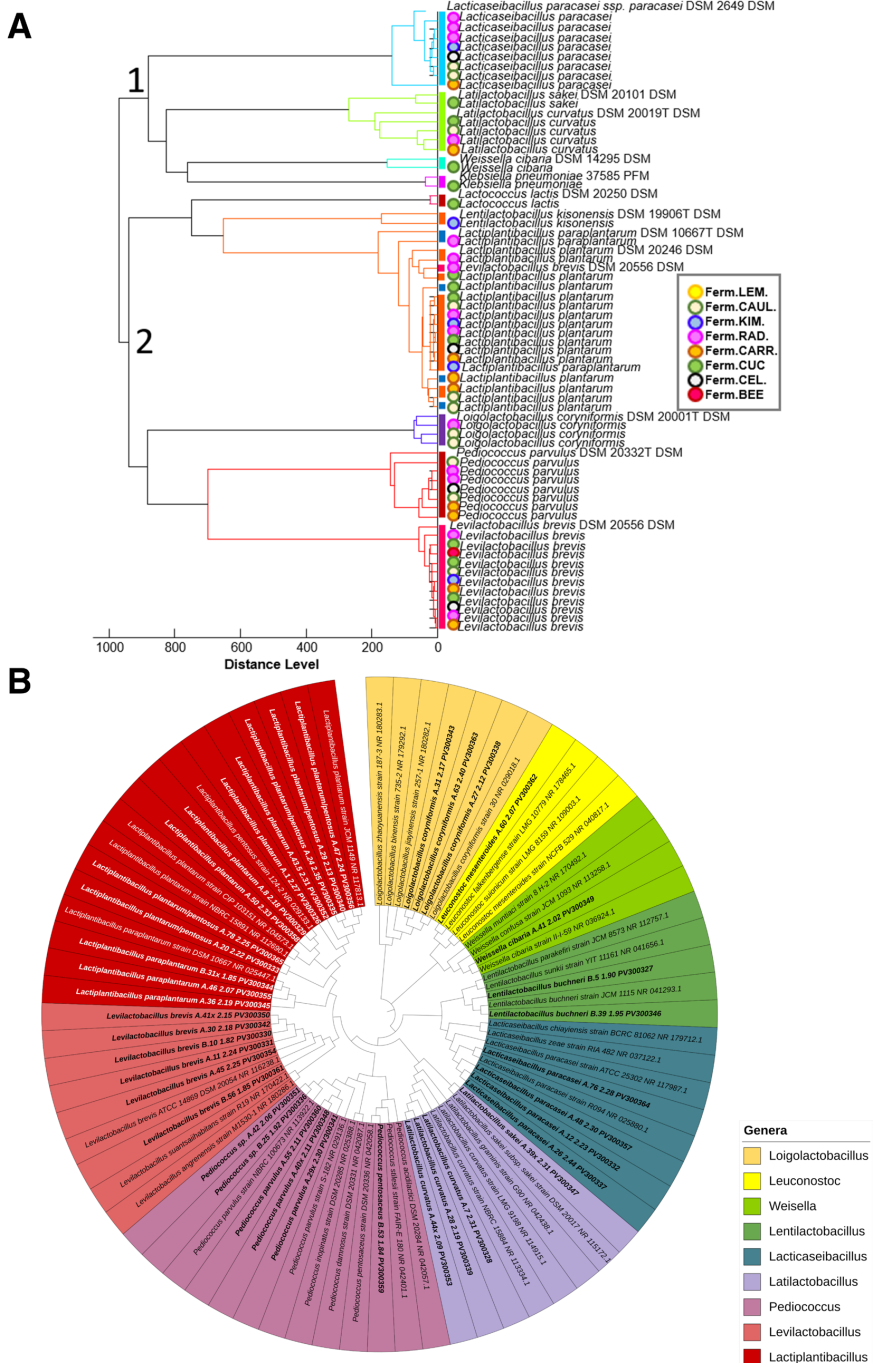
Phyloproteomic (A) and phylogenetic (B) relationships between identified bacterial isolates. The identified isolates were compared with reference strains from the MALDI Biotyper database 4.1 (A) and NCBI database (B). The source materials from which the isolates originated were marked with the corresponding symbols on the MSP. Strains deposited in the NCBI database are highlighted in bold on the phylogenetic tree. For a detailed explanation of the abbreviations used, please see Figure 1.

The phyloproteomic analysis of the identified isolates revealed the presence of two major clusters, each comprising smaller groups of closely related bacterial species (Figure 4A). The first cluster includes strains belonging to the genera *Lacticaseibacillus* and *Latilactobacillus*. The secondcluster is characterised by a large subgroup composed mainly of *Lactiplantibacillus paraplantarum* and *Lactiplantibacillus plantarum*, which were predominantly isolated from the fermented radish, cucumber and carrot. Additionally, this cluster contains a distinct subgroup formed by *Pediococcus parvulus* and *Levilactobacillus brevis*. The topology of the phylogenetic tree correlated well with the species‐specific protein profiles generated by the MALDI‐TOF MS (Figure 4B). The MSP‐based clustering showed strong concordance with the phylogenetic relationships inferred from the 16S rRNA sequence analysis, enabling the delineation of nine distinct and coherent subclusters of the isolates. In addition, the 16S rRNA gene sequencing enabled the species‐level identification of the *Pediococcus* isolates, positioning them alongside the closely related reference strains of *Pediococcus parvulus* .

The biodiversity indices (the Shannon Index and Simpson Index) were calculated to compare the microbial diversity across fermented food types (Figure S2). The Kruskal–Wallis test indicated a statistically significant difference in the microbial diversity across the different types of fermented products (*p* < 0.01). A higher Shannon–Wiener Index was observed for the fermented cauliflower (2.83), radish (2.52) and cucumber2 (2.30), confirming a greater microbial diversity and a more balanced distribution of microorganisms in these fermentation products. In addition, the Shannon–Wiener Index differed significantly between the cauliflower and the cucumber (*p* = 0.046) as well as between the cucumber and the radish (*p* = 0.004). The lowest Shannon Index was recorded for the fermented lemon (0.00) and beetroot (1.10) (Figure S2A). The greater diversity observed in the cauliflower, radish, and cucumber2 was further supported by high Simpson Index values (0.94, 0.91 and 0.90, respectively) (Figure S2B).

**FIGURE 5:**
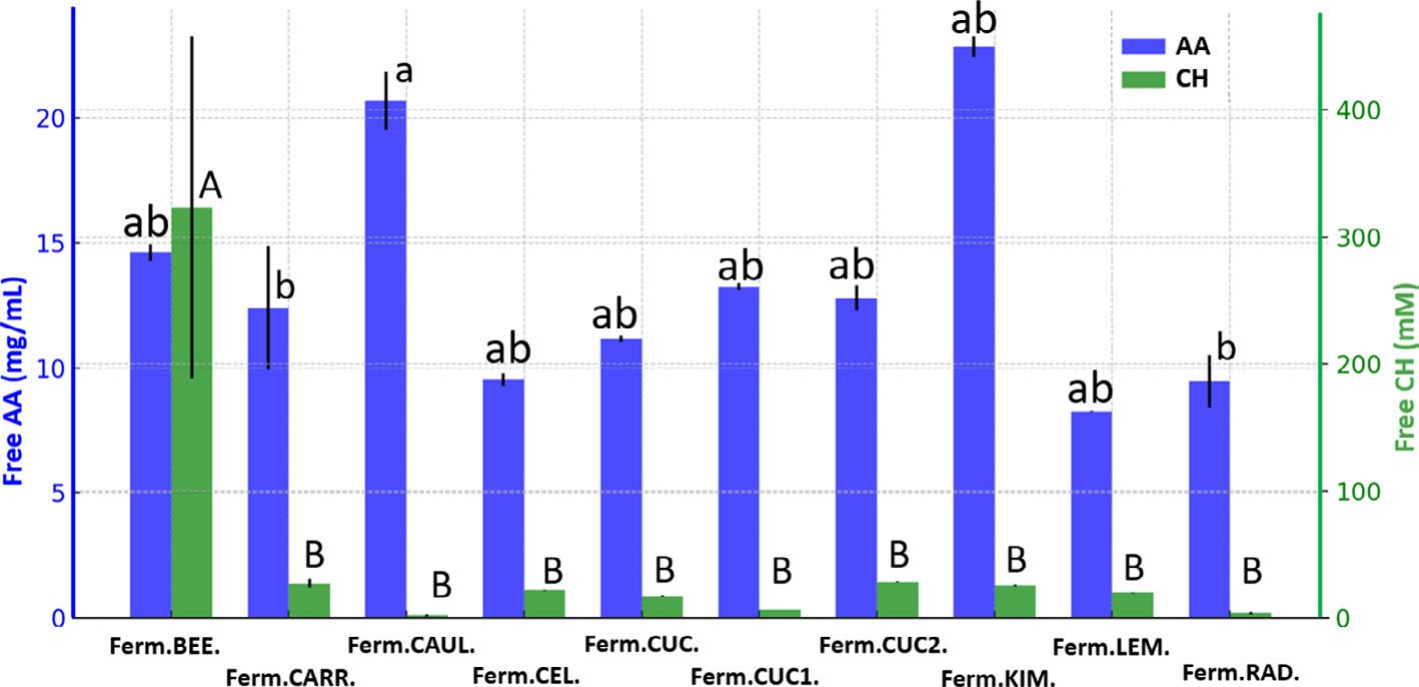
The concentrations of amino acids (AA) and carbohydrates (CH) in relation to the type of fermented plant product. Significant differences between the concentrations of free AA (lowercase letters) and free CH (capital letters) are indicated by different letters. For a detailed explanation of the abbreviations used, please see Figure 1.

The pH of the analysed fermented products ranged from 3.05 to 3.70 for the majority of plant‐based fermented matrices (Table S2). An exception was the fermented lemon, which exhibited the lowest pH value of 2.41. The Kruskal–Wallis test results indicated statistically significant differences in the concentration of amino acids (AA) and carbohydrates (CH) depending on the type of fermented food (*p* < 0.01) (Figure 5). The highest concentration of amino acids was significantly higher in the fermented cauliflower (20.67 mg/mL) than in the fermented carrot (12.39 mg/mL) and the fermented radish (9.46 mg/mL). The carbohydrate concentration was significantly higher in the fermented beetroot (323.45 mM) compared to all other fermented vegetables.

**FIGURE 6:**
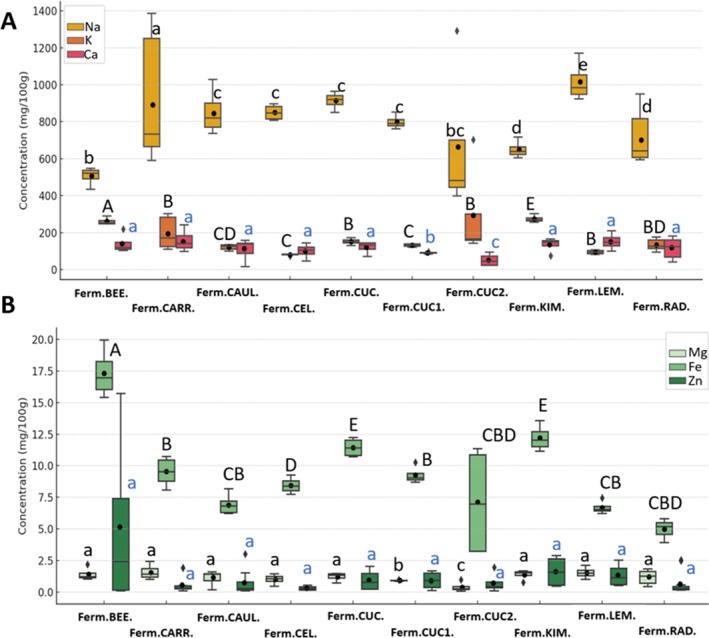
The concentrations of Na, K, and Ca (A) and Mg, Fe, and Zn (B) in relation to different fermented plant‐based matrices. Significant differences between ion concentrations are indicated by different types of letters: Lowercase letters for Na and Mg, capital letters for K and Fe and blue letters for Ca and Zn. The mean values of mineral concentrations are indicated on the graph as dots. For a detailed explanation of the abbreviations used, please see Figure 1.

Statistically significant differences were observed in the concentrations of Na, K, Ca, Mg and Fe ions depending on the type of fermented product (*p* = 0.001, *p* < 0.00002, *p* = 0.046, *p* = 0.037 and *p* < 0.001, respectively) (Figure 6). In contrast, the Zn concentration did not differ significantly across the fermented samples. The highest sodium concentration was recorded in the fermented carrot (891.72 mg/100 g), which was significantly higher than in all other fermented products. The sodium concentrations did not differ significantly between the cauliflower, celery, and two types of fermented cucumbers, with mean values of 844.56, 850.20, 914.09 and 799.14 mg/100 g, respectively. The lowest Na concentration was found in the fermented beetroot (506.91 mg/100 g). The highest potassium concentration was observed in the fermented beetroot (261.43 mg/100 g), which was significantly higher than in all other samples. The K concentration was comparable in the fermented carrot, two types of cucumbers, lemon and radish, with mean values of 152.79, 152.65, 132.84, 140.43 and 133.50 mg/100 g, respectively. The highest calcium concentration was recorded in the fermented carrot (152.80 mg/100 g) and was significantly higher than in the cucumber1 (90.89 mg/100 g) and cucumber2 (52.56 mg/100 g) (Figure 6A). The highest magnesium concentration was observed in the fermented carrot (1.58 mg/100 g); however, it was significantly higher only compared with cucumber1 (0.91 mg/100 g) and cucumber2 (0.37 mg/100 g). Among all the analysed fermented vegetables, cucumber2 exhibited the lowest Mg concentration. The highest iron concentration was recorded in the fermented beetroot (17.32 mg/100 g) and was significantly higher than in all other samples. The lowest Fe concentrations were observed in cucumber2 (7.12 mg/100 g) and radish (4.97 mg/100 g). The highest zinc concentration was observed in the fermented beetroot (5.15 mg/100 g); however, it did not differ significantly from the other studies samples (Figure 6B).

**FIGURE 7:**
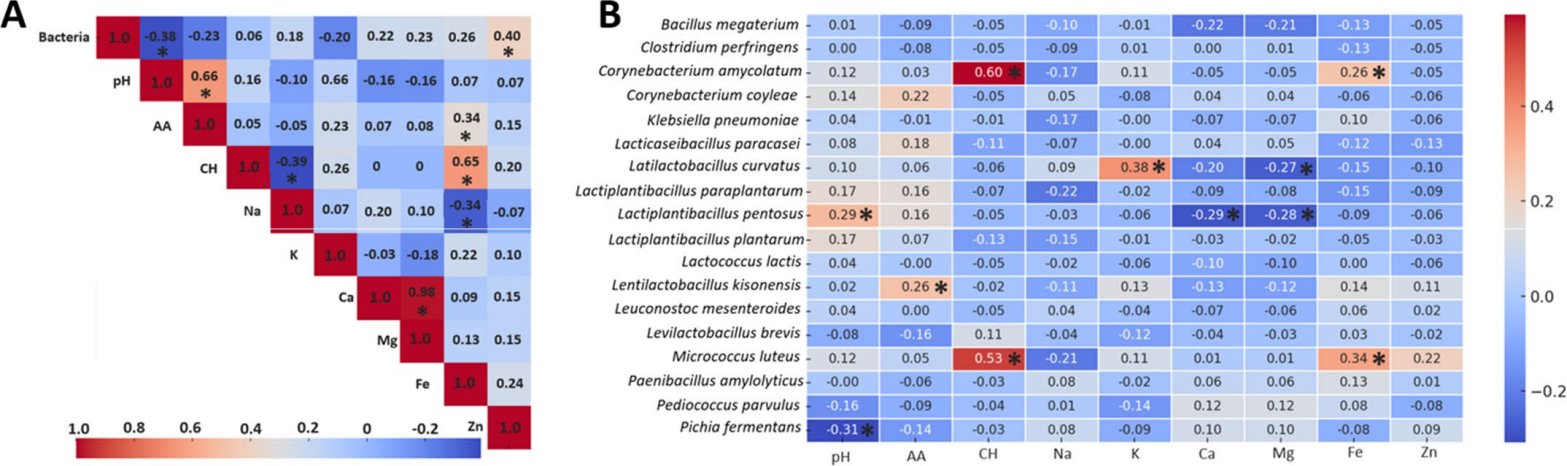
Visual representation of the Pearson's correlation coefficient (PCC) between the analysed parameters (A), including correlations for identified bacterial species (B). Statistically significant correlations (*p* < 0.05) are indicated by stars (*). AA‐ amino acids, CH‐ carbohydrates.

Several statistically significant correlations were observed between bacterial occurrence and the physicochemical parameters of the fermented vegetables (Figure 7). The presence of bacteria showed a significant negative correlation with the pH of fermented products (PCC = −0.38) and a positive correlation with the Zn ion concentration (PCC = 0.40). Strong positive and statistically significant correlations were identified between pH and free amino acids (PCC = 0.66), carbohydrates and Fe ions (PCC = 0.65), Ca and Mg ions (PCC = 0.98) (Figure 7A). A statistically significant positive correlation was observed between the occurrence of *Lactiplantibacillus pentosus* and pH, whereas its presence was negatively correlated with the Ca and Mg concentrations. Additionally, a significant positive correlation was found between *Lentilactobacillus kisonensis* and amino acids. Furthermore, the concentration of carbohydrates was positively correlated with *Corynebacterium amycolatum* and *Micrococcus luteus* , while iron ions also showed a positive correlation with these two bacterial species (Figure 7B).

The protein profiles of the three selected bacterial species (*Levilactobacillus brevis*, *Lacticaseibacillus paracasei* and *Lactiplantibacillus plantarum*) isolated from different fermented raw materials and cultured on the same growth medium were analysed and compared using the mass spectrometry spectra. The gel view of the MALDI−TOF MS spectra obtained from three bacterial species revealed differences in the protein profiles depending on the type of fermented matrices (Figure S3). The degree of the mass spectrum complexity varied depending on the type of fermented matrices from which the isolates were obtained (Figures S3 and S4). For *L. brevis* , the highest number of *m*/*z* signals in the 2000–15,500 Da range was detected when the bacteria were isolated from the cucumber2 (*N* = 91), followed by the beetroot (*N* = 80). A comparable number of *m*/*z* signals was obtained from the celery, carrot and kimchi (*N* = 69–77). In contrast, the least abundant spectral profiles were recorded for *L. brevis* isolated from the radish (*N* = 58) and cauliflower (*N* = 60). The richest MS spectrum was observed after the isolation of *L. paracasei* from the cauliflower (*N* = 88), followed by the radish (*N* = 78). The least complex spectral profile was obtained for *L. paracasei* isolated from the kimchi. For *Lactiplantibacillus plantarum*, the most abundant spectral profile was recorded for isolates obtained from cucumbers (*N* = 70–87). A similar number of *m*/*z* signals was detected after bacteria isolation from the kimchi, cauliflower, celery and carrot (*N* = 50–58).

Several differentiating *m*/*z* signals associated with specific raw plant materials were successfully identified using the UniProt database, allowing for the assignment of corresponding proteins to selected spectral signals (Table 1). For *Levilactobacillus brevis*, a signal at *m*/*z* 6975 Da, detected in isolates from the cucumber, beetroot, and cauliflower, corresponded to a bacterial ribosomal protein of the bL32 family. Another signal at *m*/*z* 7278 Da, additionally present in isolates from the carrot, was assigned to a DUF2187 domain‐containing protein. In isolates of *L. brevis* obtained from the kimchi, celery, and cucumber, an *m*/*z* 9436 Da peak corresponded to the phosphor carrier protein HPr involved in carbohydrate transport and metabolism. In the case of *Lacticaseibacillus paracasei*, a peak at *m*/*z* 4450 Da, detected in isolates from fermented radish, cauliflower, celery, and carrot, was associated with a peptide derived from the teichoic acid/polysaccharide phosphoglycerol transferase. Another signal at *m*/*z* 7440 Da was matched to a peptide fragment of an amino acid/polyamine transporter protein. For *Lactiplantibacillus plantarum*, a signal at *m*/*z* 4532 Da, observed in isolates from celery, cucumber1 and 2, carrot, and radish, was assigned to a peptide corresponding to the 50S ribosomal protein L36 of the chloroplast. Furthermore, a signal at *m*/*z* 7248 Da, present in isolates from the cauliflower, cucumber, and kimchi, was linked to a fragment of the foldase protein PrsA, while the *m*/*z* 7429 Da peak was identified as a prophage‐associated protein fragment.

**TABLE 1:** Selected spectral signals identified based on the UniProtKB database.

Bacteria species	Fermented plant materials	*m*/*z*	Intensity	UniProtKB entry	Protein names
*Levilactobacillus brevis*	Cucumber	6975	4585	A0AA41ES00	Large ribosomal subunit protein bL32
	Beetroot		20,414		
	Cauliflower		21,078		
*Levilactobacillus brevis*	Beetroot	7278	4870	A0A0R2LWP3	DUF2187 domain‐ containing protein
	Cucumber		2868		
	Cauliflower		11,154		
	Carrot		8783		
*Levilactobacillus brevis*	Kimchi	9436	22,561	A0A0R1GQD0	Phosphocarrier protein HPr
	Celery		2400		
	Cucumber		8279		
*Lacticaseibacillus paracasei*	Radish	4450	43,023	A0A829G6P9	Teichoic acid/polysaccharide phosphoglycerol transferase
	Cauliflower		19,412		
	Celery		46,131		
	Carrot		27,057		
*Lacticaseibacillus paracasei*	Cauliflower	7440	6314	A0A829G0I6	Amino acid/polyamine transporter protein
	Radish		5076		
	Carrot		8661		
*Lactiplantibacillus plantarum*	Celery	4532	6146	A0A512PKN5	Large ribosomal subunit protein bL36
	Celery		6146		
	Cucumber1		13,611		
	Carrot		10,726		
	Cucumber2		4868		
	Radish		17,121		
*Lactiplantibacillus plantarum*	Cauliflower	7248	6299	A0AB34XYT1	Foldase protein PrsA
	Cucumber		8666		
	Kimchi		4906		
*Lactiplantibacillus plantarum*	Kimchi	7429	10,230	A0AAW3FQ10	Prophage protein
	Cauliflower		15,843		
	Cucumber		16,785		

*Note:* The table presents the bacterial species and types of fermented plant materials in which isolates with *m*/*z* signals were identified, along with the corresponding signal intensities. For each *m*/*z* value, the matched UniProtKB entry and associated protein name corresponding to the peptide fragment are provided.

The NMDS analysis provided a visual representation of the relationships between mass spectral profiles of *L. brevis, L. paracasei, and L. plantarum*, depending on the type of fermented vegetables from which the isolates were obtained (Figure S5). For *L. brevis* , the mass spectral profiles of isolates from cucumber1 and radish exhibited similar patterns, as did those from beetroot and cucumber, as well as carrot, cauliflower and cucumber2. The analysis of differences in mass spectral profiles for *L. paracasei* did not reveal any similarities among the profiles obtained from isolates of the same species from different fermentation matrices. Among the mass spectral profiles of *L. plantarum* , the most closely related profiles were isolates obtained from the cucumber and celery as well as the ones obtained from the carrot and cauliflower.

## 4 Discussion

The composition and structure of the microbial communities are strongly influenced by the type of fermented product. Several factors affect these differences, including the intrinsic macro‐ and micronutrient composition of the vegetable, pH, the water content, and the presence of natural antimicrobial compounds (Jeong et al. 2013; Marco et al. 2017). The high number of isolates obtained from the fermented cauliflower and radish indicated the supporting role of some fermented products in an enriched microbial ecosystem. Cauliflower has a high surface area and a complex carbohydrate composition, which may facilitate microbial attachment and colonisation. Radish contains various bioactive compounds, including glucosinolates, which can influence microbial growth by selectively promoting or inhibiting specific bacterial taxa. The predominance of isolates identified with the high consistency species identification confirmed a well‐established microbial community with reproducible and identifiable taxa. The fermented radish and cauliflower were both shown to support diverse microbial communities dominated by lactic acid bacteria, which contribute to flavour development, acidification, and preservation (Paramithiotis et al. 2010; Liu et al. 2020). The predominant bacterial genera included *Lacticaseibacillus, Lactiplantibacillus, Latilactobacillus, Levilactobacillus*, indicating that the fermentation process promotes the growth of LAB and spore‐forming *Bacillus* species, which are commonly associated with vegetable fermentation. *Lactiplantibacillus* species, particularly *L. plantarum, L. paraplantarum*, and *L. pentosus* were identified as the most frequently isolated LAB in the fermented vegetables due to their acid tolerance and metabolic versatility (Lee et al. 2015; Rao et al. 2020). The presence of *Lentilactobacillus* and *Levilactobacillus* supports the observation that multiple *LAB* species can coexist during radish kimchi fermentation, likely due to their complementary metabolic functions (Kim et al. 2021). The probiotic potential of *Lactiplantibacillus plantarum* further enriches the functional diversity within the LAB community, contributing to both the fermentation dynamics and gut health (Wang et al. 2024). The spontaneous nature of vegetable fermentation promoted the development of complex microbial consortia comprising both homofermentative and heterofermentative lactic acid bacteria. In the present study, homofermentative species such as *L. plantarum* and *L. lactis* were identified alongside the heterofermentative LAB, including *L. paracasei* , *L. brevis* and *L. buchneri* . The co‐occurrence of these metabolic types reflects a microbial succession and functional complementarity in carbohydrate metabolism (Paramithiotis et al. 2010).

The fermented lemon exhibited the lowest microbial abundance, with only a single yeast species, *Pichia fermentans* , being identified. A low microbial diversity, particularly the lack of the LAB in the fermented lemon, may indicate a poor fermentation efficiency or aselective inhibition of bacterial growth due to environmental conditions such as high acidity, osmotic stress, or antimicrobial compounds present in the lemon (e.g., citric acid and flavonoids) (Mastuki et al. 2024). Similarly, the beetroot and kimchi also exhibited low microbial counts. The highest sugar content noticed in our study for the beetroot may promote the dominance of a few fast‐growing fermentative bacteria, leading to a lower overall diversity visible in the low value of the Shannon and Simpson index. The fermentation of beetroot is typically dominated by a few acid‐tolerant LAB species, such as *Lactiplantibacillus plantarum* and *Weissella* sp., which rapidly acidify the environment, potentially limiting the growth of other microbial taxa (Filannino et al. 2018). On the other hand, the reduction in the overall bacterial community in the kimchi can be affected by salt concentration and the presence of antimicrobial spices, such as garlic and ginger (Stoll et al. 2020). The second fungal species, *Kazachstania exigua*, was detected in the fermented carrot, indicating that yeasts may coexist with the LAB in some fermentations, potentially influencing the final product's characteristics. *Pichia fermentans and Kazachstania exigua* are known non‐Saccharomyces fermentative yeasts that may contribute to the development of aroma and flavour through the production of organic acids, alcohols and esters (Wang et al. 2023).

For most bacterial isolates, the MALDI‐TOF MS identification revealed a high level of agreement with the 16S rDNA sequencing. Even with lower score consistency, the MALDI‐TOF MS can still yield an accurate genus‐ and species‐level identification for well‐represented bacterial taxa in the reference database (Pavlovic et al. 2013). Similar results were also observed in our study for *Lactiplantibacillus paraplantarum, Lentilactobacillus buchneri* and *Pediococcus parvulus* . However, some discrepancies in the consistency of identification were observed for certain species. For some isolates of *Lentilactobacillus buchneri* and *Levilactobacillus brevis*, MALDI‐TOF MS misidentified isolates even at the genus level. The accuracy of MALDI‐TOF MS relies heavily on comprehensive and high‐quality spectral databases. Misidentification may occur if the database lacks sufficient reference spectra for certain species or if reference spectra are not well differentiated between closely related species (Ludwiczak et al. 2025). Recent taxonomic reclassifications within the *Lactobacillaceae* family may not yet be fully integrated into the MALDI reference libraries, leading to the incorrect genus‐level identification (Zheng et al. 2020). In addition, accurate differentiation can be challenging due to the low ionisation efficiency of some LAB species, resulting in weaker spectra and lower identification scores (Pavlovic et al. 2013). The essential role in the effective and precise MALDI‐TOF MS identification of spore‐forming bacteria from the genus Bacillus is the optimisation of cultivation conditions and the selection of appropriate incubation periods (Janiszewska et al. 2023). Therefore, while MALDI‐TOF MS demonstrated its efficiency as a rapid and effective tool for bacterial identification, particularly in complex environmental matrices such as fermented foods, the limitations of this technique underscore the need for an integrated approach. Combining proteomic and genomic methods is essential to enhance the identification accuracy in the LAB classification. The strong agreement between the MSP‐based phyloproteomic clustering and the 16S rRNA‐based phylogenetic analysis confirmed that MALDI‐TOF MS is a reliable tool for differentiating closely related bacterial species in complex microbial communities (Pomastowski et al. 2019). The coherent taxonomic and proteomic grouping reflects both genetic similarity and protein expression profiles suggesting that protein‐level data can reflect evolutionary relationships among lactic acid bacteria. The positioning of *Pediococcus* isolates near the reference *P. parvulus* strains in both proteomic and genetic analyses confirms the complementary utility of combining MALDI‐TOF MS and 16S rRNA sequencing for resolving the taxonomically ambiguous identification. Importantly, the consistency of the microbial profiles and the MALDI‐TOF MS protein patterns across the replicate fermentations of each matrix indicates that our fixed‐matrix approach yielded reproducible results, despite the inherent complexity of spontaneous fermentation.

The concentration of carbohydrates, amino acids and some mineral components differed among selected fermented vegetables, highlighting the complex interactions between microbial communities, nutrient availability and fermentation conditions. High microbial diversity and the distribution of microbial species observed for the fermented cauliflower correlate with their higher amino acid concentrations. The fermentation substrates rich in carbohydrates and proteins support a more diverse microbial community by providing multiple metabolic pathways for microbial growth (Filannino et al. 2018). In addition, the positive correlation between the pH and the free amino acid concentration suggests that microbial activity contributes to protein hydrolysis and amino acid release during fermentation. The previous research shows that LAB species are capable of producing proteolytic enzymes, which break down proteins into free amino acids, enhancing the nutritional profile of fermented foods (Zhang et al. 2021). The low microbial diversity and the highest carbohydrate concentration noticed for the fermented beetroot may indicate less efficiency in the fermentation of carbohydrates possibly due to the dominance of fewer bacterial species with limited metabolic diversity. The strong positive correlation between carbohydrates and iron ions concentration indicated the dependence of sugar metabolism on Fe availability, potentially impacting microbial growth and diversity. The fermentation efficiency and microbial diversity in kimchi fermentations are strongly dependent on trace mineral availability (Jeong et al. 2013). The mineral composition of fermented vegetables varied significantly especially for Na, K and Fe and may have resulted from the intrinsic mineral composition of the raw materials, which is further modified by microbial metabolism during fermentation (Adebo et al. 2022). The observed negative correlation between bacterial presence and pH aligns with the expected role of LAB in acidification, which is crucial for fermentation‐driven preservation. Additionally, *Lactobacillus pentosus* was positively correlated with pH but negatively correlated with Ca and Mg concentrations. The members of the *Lactiplantibacillus* sp. and *Latilactobacillus* sp. may thrive in environments with higher pH but lower Ca and Mg ions availability due to species‐specific ion transport mechanisms that influence metabolic activity (Kleerebezem et al. 2010). Furthermore, the presence of *Corynebacterium amycolatum* and *Micrococcus luteus* was positively correlated with carbohydrate concentration and Fe ion availability. The adaptation to sugar‐rich environments and iron‐dependent metabolic pathways allows for the presence of these species in the fermented products (Mrvčić et al. 2012).

The results of the study demonstrated that the fermentation matrices' composition significantly influences the proteomic profile of the tested LAB species, with differences observed even within the same bacterial species. The environmental factors, nutrient availability, and bacterial adaptation strategies affect protein signatures, leading to variability in the mass spectral complexity and species‐specific metabolic adaptations (Papadimitriou et al. 2016). The NMDS analysis further supported the obtained results, revealing distinct clustering patterns for mass spectral profiles, depending on the fermentation matrices' composition. The *LAB* can develop strain‐specific adaptive mechanisms to improve the domestication process in artificial habitats, such as fermented foods (Mejía‐Caballero et al. 2025). The richness of signals in spectral profiles observed for some LAB species from specific types of fermentation matrices suggests that fermentation conditions may have favoured a broader range of metabolic activities, leading to a more diverse proteomic profile. *Lactiplantibacillus plantarum* exhibits broad metabolic flexibility, enabling it to adapt to various plant‐based fermentation substrates by modifying its metabolic pathways and stress response mechanisms (Paramithiotis 2025). UniProt database allows the identification of specific *m*/*z* peptide signals associated with plant‐derived matrices in individual LAB strains. A conserved DUF2187 domain‐containing protein was identified as potentially involved in the stress response or cellular regulatory mechanisms (Huang et al. 2023). Its detection in *Levilactobacillus brevis* isolates suggests a possible functional role in environmental adaptability, particularly in response to the dynamic and variable conditions of vegetable fermentation. In addition, the presence of a fragment of an amino acid/polyamine transporter protein (*m*/*z* 7440 Da) in *L. paracasei* isolates highlights the important role of transport systems in nutrient acquisition and ecological fitness in plant‐based fermentation environments (Jack et al. 2000). The present study indicates that mass spectral protein profiles are linked with the availability of fermentable carbohydrates, which plays acrucial role in bacterial metabolism. The detection of phosphocarrier protein HPr peptide fragments (*m*/*z* 9436 Da) in *L. brevis* supports an enhanced capacity for carbohydrate transport and phosphorylation (Ye and Saier 1995). In addition, the high concentration of carbohydrates in the fermented beetroot may explain the high MS complexity of *L. brevis* (Gänzle 2015). Furthermore, salt concentration and pH levels can impact bacterial stress responses and protein profiles. In addition, the upregulation of stress‐related proteins in response to high‐salt or low‐pH environments can lead to changes in MS spectral complexity (Papadimitriou et al. 2016). Therefore, to fully elucidate the relationships between the physicochemical composition of plant‐based fermentation matrices and the proteomic responses of LAB, further research is necessary. In particular, the functional validation of proteins identified in the mass spectral profiles would be essential to clarify their specific roles in LAB physiology, especially in relation to stress adaptation and metabolic activity during the fermentation process.

## 5 Conclusion

The composition of plant‐based fermentation matrices plays a crucial role in shaping the microbial community structure and dynamics, underscoring the importance of considering substrate characteristics during fermentation optimisation and microbial identification. The complex interactions between microbial communities and fermentation matrices lead to species‐specific variations in the protein MS‐profile. The protein profiles generated by MALDI‐TOF MS reflect underlying phylogenetic relationships, supporting their application in microbial ecology, taxonomy, and quality control in fermented food microbiota studies. The matrices‐dependent protein expression patterns observed in the study reflect differential metabolic activity and stress responses during the fermentation process acting on the inconsistencies in bacterial identification by MALDI‐TOF MS. It highlights the need to consider matrices‐specific proteomic signatures in the MALDI‐TOF MS databases and to supplement identification approaches with complementary genetic methods, such as 16S rRNA sequencing, especially in studies involving complex, natural fermentation systems.

## Author Contributions

**Agnieszka Ludwiczak:** conceptualization, investigation, writing – original draft, funding acquisition, methodology, visualization, validation, writing – review and editing, software, formal analysis, project administration, supervision, data curation. **Ewelina Sibińska:** investigation, visualization, writing – review and editing, methodology. **Iwona Adamczyk:** investigation, methodology, writing – review and editing. **Miłosz Wasicki:** investigation, writing – review and editing. **Oleksandra Pryshchepa:** investigation, writing – review and editing. **Michał Złoch:** visualization, writing – review and editing, investigation. **Klaudia Grygorowicz:** investigation. **Małgorzata Szultka‐Młyńska:** writing – review and editing. **Paweł Pomastowski:** conceptualization, resources, formal analysis, writing – review and editing, methodology.

## Conflicts of Interest

The authors declare no conflicts of interest.

## Supporting information


**Data S1:** emi470237‐sup‐0001‐Supinfo.docx.

## Data Availability

The datasets generated during this study are publicly available in the Zenodo repository under DOI https://doi.org/10.5281/zenodo.17358401. The sequence data of the identified species have been deposited in the GenBank database, and are publicly accessible via the following URL: https://www.ncbi.nlm.nih.gov/nuccore?term=PV300326+%3A+PV300365%5Baccn%5D&cmd=DetailsSearch&log$=activity.
