# Supplementary material for: Integration of MALDI‐TOF MS and 16S rRNA Analysis for Identification of Plant‐Based Fermentation‐Associated Microbiota
Source: Environ Microbiol Rep. 2026 Jan 27;18(1):e70237. doi: 10.1111/1758-2229.70237 (PMC12835767; doi:10.1111/1758-2229.70237)
Supplement: Supplementary file 1 — Data S1: emi470237‐sup‐0001‐Supinfo.docx. [file EMI4-18-e70237-s001.docx]

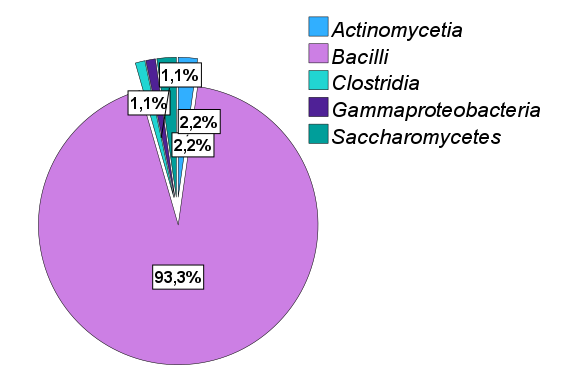


Supplementary Figure 1. Percentage distribution of identified classes in plant-based fermented materials.


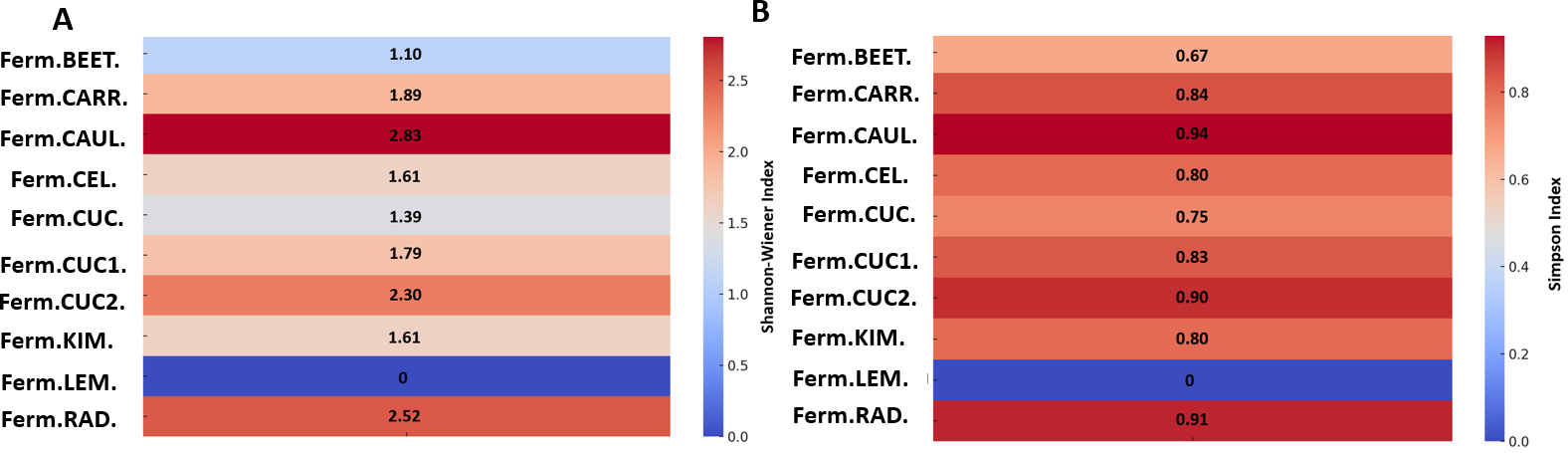


Supplementary Figure 2. Heatmaps illustrated the Shannon Index (A) and Simpson Index (B). The colour intensity corresponds to the index values. Fermented materials: Ferm.BEET- fermented beetroot, Ferm.CARR.- fermented carrot, Ferm.CAUL.- fermented cauliflower, Ferm.CEL.- fermented celery, Ferm.CUC.- fermented cucumber, Ferm.CUC1.- fermented cucumber1, Ferm.CUC2.- fermented cucumber2, Ferm.KIM.- fermented kimchi, Ferm.LEM.- fermented lemon, Ferm.RAD.- fermented radish.


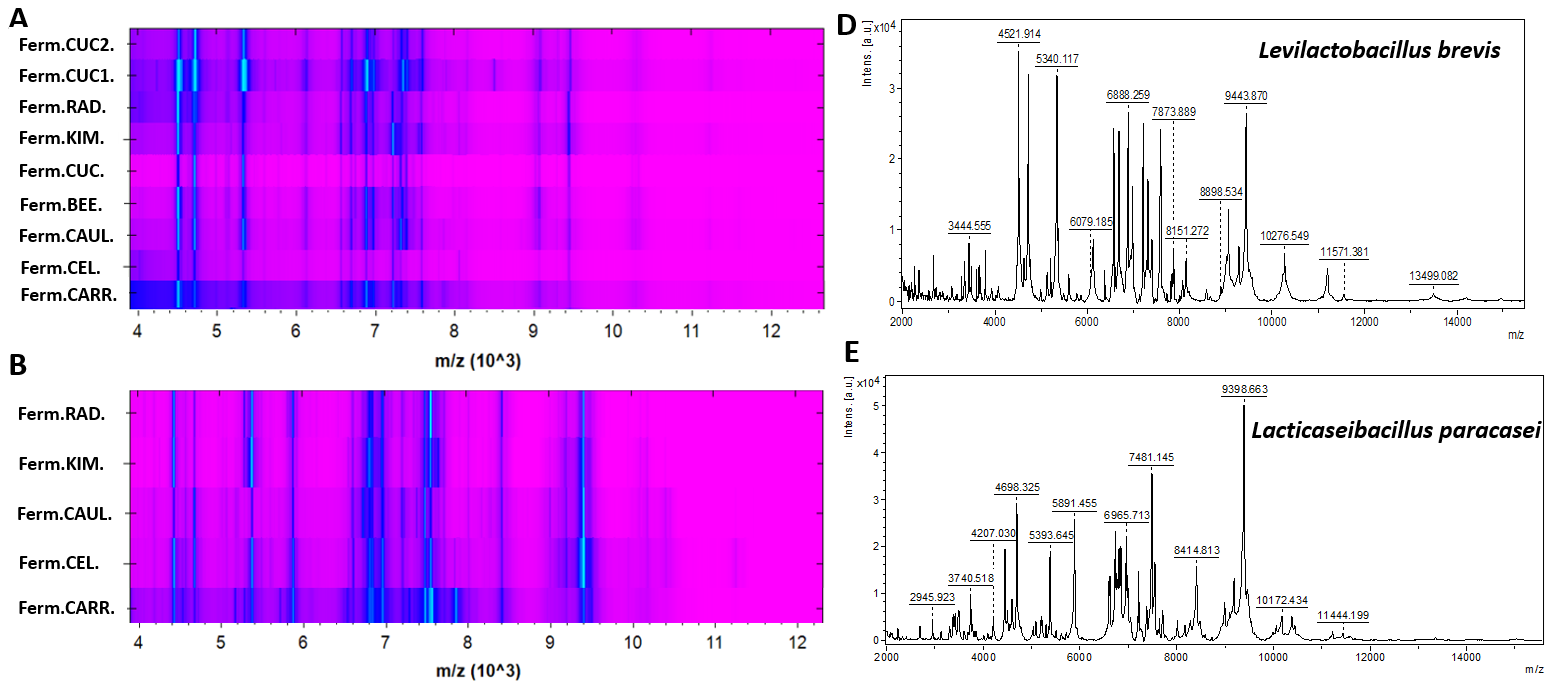


**
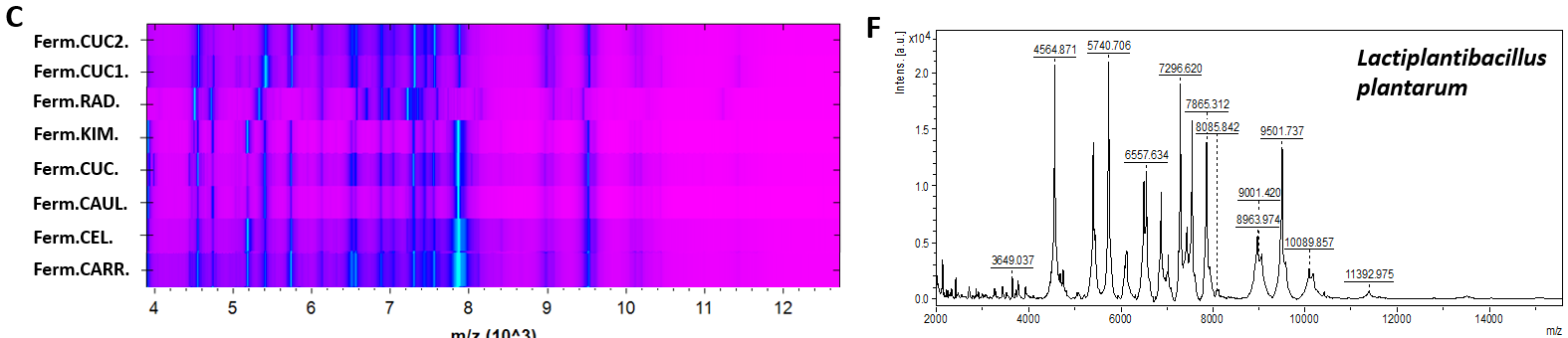
**

Supplementary Figure 3**.** MALDI-TOF MS gel view (A, B, C) and mass spectra (D, E, F) of *Levilactobacillus brevis, Lacticaseibacillus paracasei,* and *Lactobacillus plantarum* isolated from different types of plant-based fermented matrices. For a detailed explanation of the abbreviations used, please see Supp. Fig. 2.

**
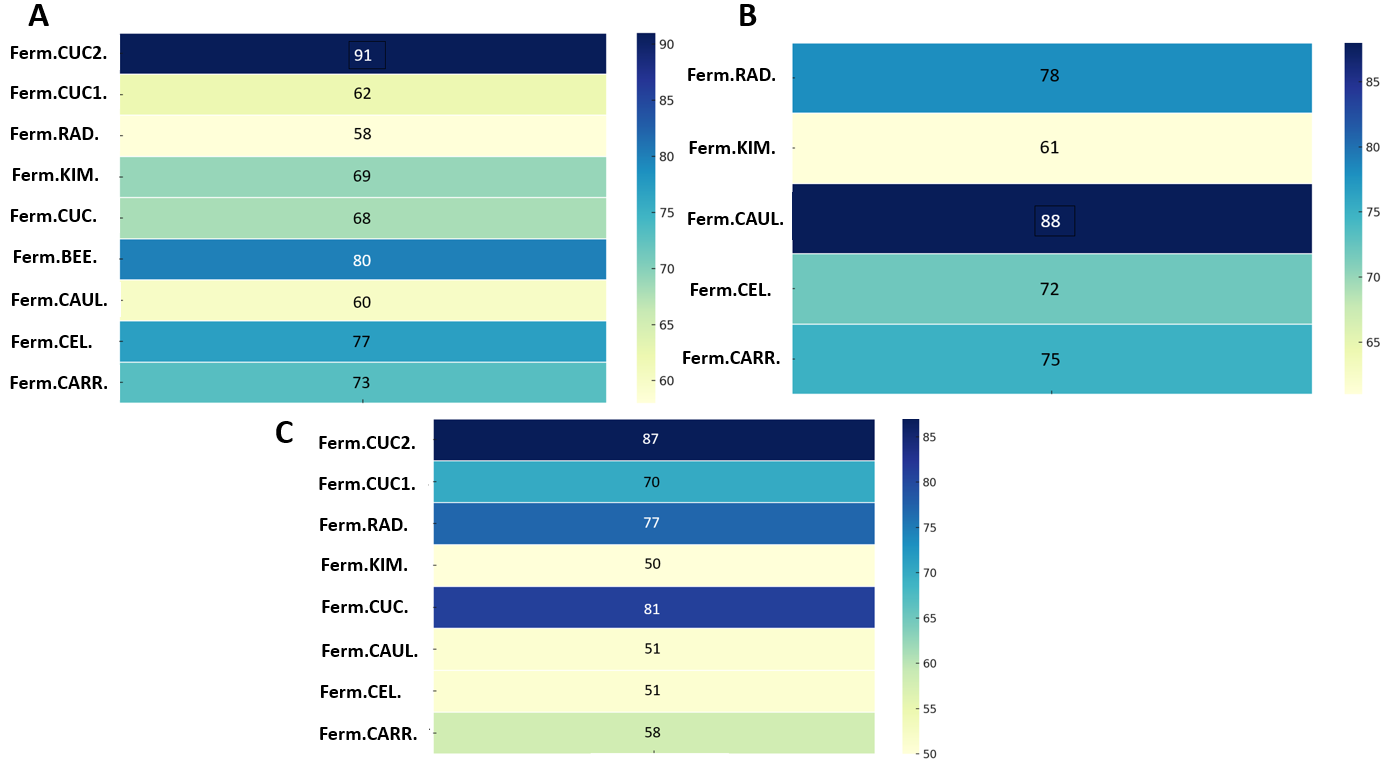
**

Supplementary Figure 4**.** Heatmap representation of the number of m/z signals in the different types of fermented vegetables for *Levilactobacillus brevis* (A), *Lacticaseibacillus paracasei* (B), and *Lactobacillus plantarum* (C). Colour intensity corresponds to the number of signals in the spectral profile. For a detailed explanation of the abbreviations used, please see Supp. Fig. 2.

**
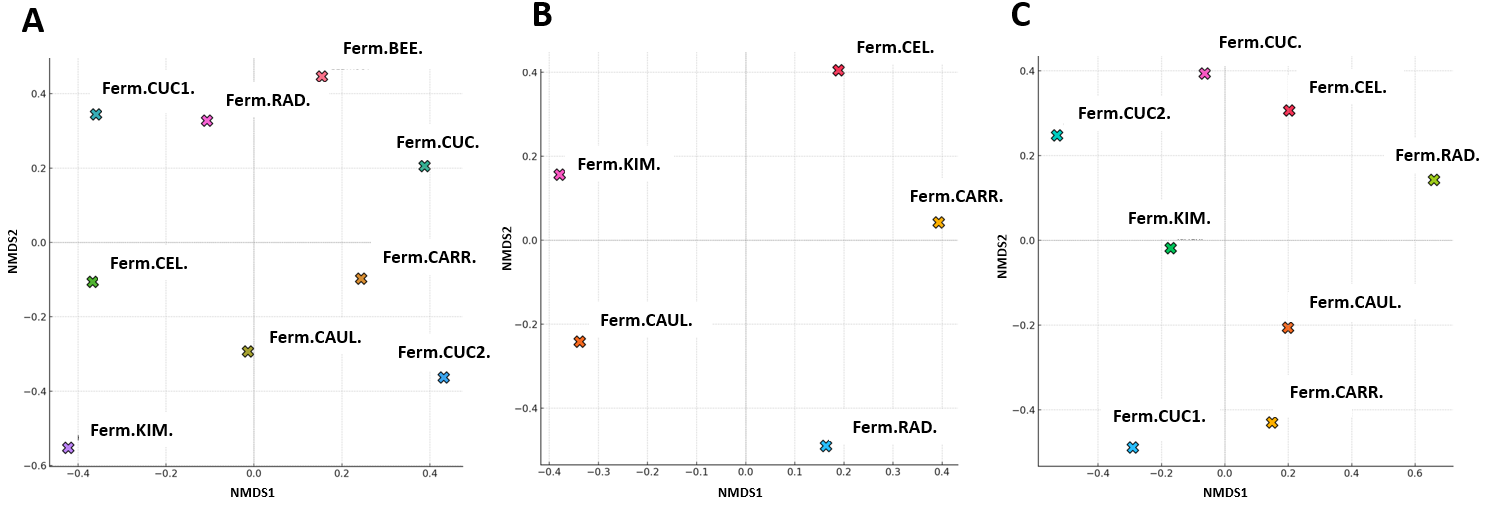
**

Supplementary Figure 5. Nonmetric multidimensional scaling (NMDS) plots representing the positioning of fermented vegetable types, determined by the non-metric scaling of dissimilarities in mass spectra (MS) profiles obtained for *Levilactobacillus brevis* (A), *Lacticaseibacillus paracasei* (B), and *Lactobacillus plantarum* (C). Each type of fermented vegetable is marked by 'x', representing an aggregated profile. The relative positions reflect their similarities, based on the Bray-Curtis distance of their m/z and intensity profiles. For a detailed explanation of the abbreviations used, please see Supp. Fig. 2.

Supplementary Table 1. The results of bacterial identification by MALDI and 16S rDNA sequencing were presented after comparing the sequences with the most related sequences available in the NCBI database, along with the indicated level of identity.

| **MALDI identification** | **Score value** | **Consistency** | **Related species from NCBI**  **[Accesion number]** | **Identity**  **[%]** | **Accesion number** |
| --- | --- | --- | --- | --- | --- |
| *Lacticaseibacillus paracasei* | 2.30 | A | *Lacticaseibacillus paracasei strain R094* | 99.86 | [PV300357](https://www.ncbi.nlm.nih.gov/nuccore/PV300357) |
|  |  |  | *Lacticaseibacillus chiayiensis strain BCRC 81062* | 99.04 |  |
|  |  |  | *Lacticaseibacillus zeae strain RIA 482* | 99.04 |  |
| *Lacticaseibacillus paracasei* | 2.28 | A | *Lacticaseibacillus paracasei strain R094* | 99.79 | [PV300364](https://www.ncbi.nlm.nih.gov/nuccore/PV300364) |
|  |  |  | *Lacticaseibacillus chiayiensis strain BCRC 81062* | 99.03 |  |
|  |  |  | *Lacticaseibacillus zeae strain RIA 482* | 99.03 |  |
| *Lacticaseibacillus paracasei* | 2.44 | A | *Lacticaseibacillus paracasei strain ATCC 25302* | 99.65 | [PV300337](https://www.ncbi.nlm.nih.gov/nuccore/PV300337) |
|  |  |  | *Lacticaseibacillus chiayiensis strain BCRC 81062* | 98.77 |  |
|  |  |  | *Lacticaseibacillus zeae strain RIA 482* | 98.77 |  |
| *Lacticaseibacillus paracasei* | 2.23 | A | *Lacticaseibacillus paracasei strain ATCC 25302* | 99.79 | [PV300332](https://www.ncbi.nlm.nih.gov/nuccore/PV300332) |
|  |  |  | *Lacticaseibacillus chiayiensis strain BCRC 81062* | 98.70 |  |
|  |  |  | *Lacticaseibacillus zeae strain RIA 482* | 98.70 |  |
| *Latilactobacillus*  *curvatus* | 2.31 | A | *Latilactobacillus curvatus strain NBRC 15884* | 99.86 | [PV300328](https://www.ncbi.nlm.nih.gov/nuccore/PV300328) |
|  |  |  | *Latilactobacillus graminis strain G90 (1)* | 99.52 |  |
|  |  |  | *Latilactobacillus sakei subsp. sakei strain DSM 20017* | 99.20 |  |
| *Latilactobacillus*  *curvatus* | 2.19 | A | *Latilactobacillus curvatus strain LMG 9198* | 99.36 | [PV300339](https://www.ncbi.nlm.nih.gov/nuccore/PV300339) |
|  |  |  | *Latilactobacillus graminis strain G90 (1)* | 98.71 |  |
|  |  |  | *Latilactobacillus sakei subsp. sakei strain DSM 20017* | 98.64 |  |
| *Lactiplantibacillus paraplantarum* | 2.07 | A | *Lactiplantibacillus paraplantarum strain DSM 10667* | 99.93 | [PV300355](https://www.ncbi.nlm.nih.gov/nuccore/PV300355) |
|  |  |  | *Lactiplantibacillus plantarum strain CIP 103151* | 99.72 |  |
|  |  |  | *Lactiplantibacillus pentosus strain 124-2* | 99.72 |  |
| *Lactiplantibacillus sp.* | 1.85 | B | *Lactiplantibacillus paraplantarum strain DSM 10667* | 99.66 | [PV300344](https://www.ncbi.nlm.nih.gov/nuccore/PV300344) |
|  |  |  | *Lactiplantibacillus plantarum strain JCM 1149* | 99.52 |  |
|  |  |  | *Lactiplantibacillus pentosus strain 124-2* | 99.45 |  |
| *Lactiplantibacillus paraplantarum* | 2.19 | A | *Lactiplantibacillus paraplantarum strain DSM 10667* | 100.00 | [PV300345](https://www.ncbi.nlm.nih.gov/nuccore/PV300345) |
|  |  |  | *Lactiplantibacillus plantarum strain CIP 103151* | 99.79 |  |
|  |  |  | *Lactiplantibacillus pentosus strain 124-2* | 99.79 |  |
| *Lactiplantibacillus plantarum* | 2.18 | A | *Lactiplantibacillus plantarum strain JCM 1149* | 99.59 | [PV300329](https://www.ncbi.nlm.nih.gov/nuccore/PV300329) |
|  |  |  | *Lactiplantibacillus pentosus strain 124-2* | 99.45 |  |
|  |  |  | *Lactiplantibacillus paraplantarum strain DSM 10667* | 99.25 |  |
| *Lactiplantibacillus plantarum* | 2.22 | A | *Lactiplantibacillus plantarum strain CIP 103151* | 99.58 | [PV300333](https://www.ncbi.nlm.nih.gov/nuccore/PV300333) |
|  |  |  | *Lactiplantibacillus pentosus strain 124-2* | 99.58 |  |
|  |  |  | *Lactiplantibacillus paraplantarum strain DSM 10667* | 99.45 |  |
| *Lactiplantibacillus plantarum* | 2.33 | A | *Lactiplantibacillus plantarum strain JCM 1149* | 99.86 | [PV300358](https://www.ncbi.nlm.nih.gov/nuccore/PV300358) |
|  |  |  | *Lactiplantibacillus pentosus strain 124-2* | 99.79 |  |
|  |  |  | *Lactiplantibacillus paraplantarum strain DSM 10667* | 99.59 |  |
| *Lactiplantibacillus plantarum* | 2.13 | A | *Lactiplantibacillus plantarum strain CIP 103151* | 99.86 | [PV300340](https://www.ncbi.nlm.nih.gov/nuccore/PV300340) |
|  |  |  | *Lactiplantibacillus pentosus strain 124-2* | 99.86 |  |
|  |  |  | *Lactiplantibacillus paraplantarum strain DSM 10667* | 99.72 |  |
| *Lactiplantibacillus plantarum* | 2.25 | A | *Lactiplantibacillus plantarum strain CIP 103151* | 99.93 | [PV300365](https://www.ncbi.nlm.nih.gov/nuccore/PV300365) |
|  |  |  | *Lactiplantibacillus pentosus strain 124-2* | 99.93 |  |
|  |  |  | *Lactiplantibacillus paraplantarum strain DSM 10667* | 99.79 |  |
| *Lactiplantibacillus plantarum* | 2.31 | A | *Lactiplantibacillus plantarum strain NBRC 15891* | 99.86 | [PV300352](https://www.ncbi.nlm.nih.gov/nuccore/PV300352) |
|  |  |  | *Lactiplantibacillus pentosus strain 124-2* | 99.72 |  |
|  |  |  | *Lactiplantibacillus paraplantarum strain DSM 10667* | 99.58 |  |
| *Lactiplantibacillus plantarum* | 2.14 | A | *Lactiplantibacillus plantarum strain JCM 1149* | 99.59 | [PV300334](https://www.ncbi.nlm.nih.gov/nuccore/PV300334) |
|  |  |  | *Lactiplantibacillus pentosus strain 124-2* | 99.58 |  |
|  |  |  | *Lactiplantibacillus paraplantarum strain DSM 10667* | 99.45 |  |
| *Lactiplantibacillus plantarum* | 2.35 | A | *Lactiplantibacillus plantarum strain CIP 103151* | 99.72 | [PV300335](https://www.ncbi.nlm.nih.gov/nuccore/PV300335) |
|  |  |  | *Lactiplantibacillus pentosus strain 124-2* | 99.72 |  |
|  |  |  | *Lactiplantibacillus paraplantarum strain DSM 10667* | 99.59 |  |
| *Lactiplantibacillus plantarum* | 2.27 | A | *Lactiplantibacillus plantarum strain NBRC 1589* | 99.72 | [PV300326](https://www.ncbi.nlm.nih.gov/nuccore/PV300326) |
|  |  |  | *Lactiplantibacillus pentosus strain 124-2* | 99.59 |  |
|  |  |  | *Lactiplantibacillus paraplantarum strain DSM 10667* | 99.45 |  |
| *Lactiplantibacillus plantarum* | 2.24 | A | *Lactiplantibacillus plantarum strain CIP 103151* | 99.86 | [PV300356](https://www.ncbi.nlm.nih.gov/nuccore/PV300356) |
|  |  |  | *Lactiplantibacillus pentosus strain 124-2* | 99.86 |  |
|  |  |  | *Lactiplantibacillus paraplantarum strain DSM 10667* | 99.65 |  |
| *Latilactobacillus sakei* | 2.31 | A | *Latilactobacillus sakei subsp. sakei strain DSM 20017* | 99.86 | [PV300347](https://www.ncbi.nlm.nih.gov/nuccore/PV300347) |
|  |  |  | *Latilactobacillus curvatus strain LMG 9198* | 98.86 |  |
|  |  |  | *Latilactobacillus graminis strain G90 (1)* | 98.84 |  |
| *Lentilactobacillus sp.* | 1.90 | B | *Lentilactobacillus buchneri strain JCM 1115* | 99.04 | [PV300327](https://www.ncbi.nlm.nih.gov/nuccore/PV300327) |
|  |  |  | *Lentilactobacillus sunkii strain YIT 11161* | 98.28 |  |
|  |  |  | *Lentilactobacillus parakefiri strain JCM 8573* | 98.28 |  |
| *Lentilactobacillus sp.* | 1.95 | B | *Lentilactobacillus buchneri strain JCM 1115* | 99.52 | [PV300346](https://www.ncbi.nlm.nih.gov/nuccore/PV300346) |
|  |  |  | *Lentilactobacillus sunkii strain YIT 11161* | 98.76 |  |
|  |  |  | *Lentilactobacillus parakefiri strain JCM 8573* | 98.76 |  |
| *Leuconostoc mesenteroides* | 2.07 | A | *Leuconostoc mesenteroides subsp. dextranicum strain NCFB 529* | 99.79 | [PV300362](https://www.ncbi.nlm.nih.gov/nuccore/PV300362) |
|  |  |  | *Leuconostoc falkenbergense strain LMG 10779* | 99.58 |  |
|  |  |  | *Leuconostoc suionicum strain LMG 8159* | 99.51 |  |
| *Levilactobacillus sp.* | 1.82 | B | *Levilactobacillus brevis ATCC 14869 = DSM 20054* | 99.72 | [PV300330](https://www.ncbi.nlm.nih.gov/nuccore/PV300330) |
|  |  |  | *Levilactobacillus angrenensis strain M1530-1* | 98.74 |  |
|  |  |  | *Levilactobacillus suantsaiihabitans strain R19* | 98.49 |  |
| *Levilactobacillus brevis* | 2.25 | A | *Levilactobacillus brevis ATCC 14869 = DSM 20054* | 99.93 | [PV300354](https://www.ncbi.nlm.nih.gov/nuccore/PV300354) |
|  |  |  | *Levilactobacillus angrenensis strain M1530-1* | 98.95 |  |
|  |  |  | *Levilactobacillus suantsaiihabitans strain R19* | 98.69 |  |
| *Levilactobacillus brevis* | 2.24 | A | *Levilactobacillus brevis ATCC 14869 = DSM 20054* | 99.86 | [PV300331](https://www.ncbi.nlm.nih.gov/nuccore/PV300331) |
|  |  |  | *Levilactobacillus angrenensis strain M1530-1* | 98.88 |  |
|  |  |  | *Levilactobacillus suantsaiihabitans strain R19* | 98.56 |  |
| *Levilactobacillus brevis* | 2.18 | A | *Levilactobacillus brevis ATCC 14869 = DSM 20054* | 99.93 | [PV300342](https://www.ncbi.nlm.nih.gov/nuccore/PV300342) |
|  |  |  | *Levilactobacillus angrenensis strain M1530-1* | 98.95 |  |
|  |  |  | *Levilactobacillus suantsaiihabitans strain R19* | 98.49 |  |
| *Levilactobacillus brevis* | 2.15 | A | *Levilactobacillus brevis ATCC 14869 = DSM 20054* | 99.86 | [PV300350](https://www.ncbi.nlm.nih.gov/nuccore/PV300350) |
|  |  |  | *Levilactobacillus angrenensis strain M1530-1* | 98.61 |  |
|  |  |  | *Levilactobacillus suantsaiihabitans strain R19* | 98.23 |  |
| *Levilactobacillus sp.* | 1.85 | B | *Levilactobacillus brevis ATCC 14869 DSM 20054* | 99.93 | [PV300361](https://www.ncbi.nlm.nih.gov/nuccore/PV300361) |
|  |  |  | *Levilactobacillus angrenensis strain M1530-1* | 98,95 |  |
|  |  |  | *Levilactobacillus suantsaiihabitans strain R19* | 98.74 |  |
| *Loigolactobacillus coryniformis* | 2.40 | A | *Loigolactobacillus coryniformis subsp. torquens strain 30* | 99.79 | [PV300363](https://www.ncbi.nlm.nih.gov/nuccore/PV300363) |
|  |  |  | *Loigolactobacillus jiayinensis strain 257-1* | 99.10 |  |
|  |  |  | *Loigolactobacillus binensis strain 735-2* | 98.95 |  |
| *Loigolactobacillus coryniformis* | 2.12 | A | *Loigolactobacillus coryniformis subsp. torquens strain 30* | 99.73 | [PV300338](https://www.ncbi.nlm.nih.gov/nuccore/PV300338) |
|  |  |  | *Loigolactobacillus jiayinensis strain 257-1* | 99.03 |  |
|  |  |  | *Loigolactobacillus binensis strain 735-2* | 98.88 |  |
| *Loigolactobacillus coryniformis* | 2.17 | A | *Loigolactobacillus coryniformis subsp. torquens strain 30* | 99.52 | [PV300343](https://www.ncbi.nlm.nih.gov/nuccore/PV300343) |
|  |  |  | *Loigolactobacillus jiayinensis strain 257-1* | 99.23 |  |
|  |  |  | *Loigolactobacillus zhaoyuanensis strain 187-3* | 98.95 |  |
| *Pediococcus sp.* | 1.84 | B | *Pediococcus pentosaceus strain DSM 20336* | 99.19 | [PV300359](https://www.ncbi.nlm.nih.gov/nuccore/PV300359) |
|  |  |  | *Pediococcus stilesii strain FAIR-E 180* | 97.83 |  |
|  |  |  | *Pediococcus acidilactici DSM 20284* | 97,49 |  |
| *Pediococcus parvulus* | 2.30 | A | *Pediococcus parvulus strain NBRC 100673* | 99.79 | [PV300341](https://www.ncbi.nlm.nih.gov/nuccore/PV300341) |
|  |  |  | *Pediococcus damnosus strain DSM 20331* | 98.63 |  |
|  |  |  | *Pediococcus inopinatus strain DSM 20285* | 98.56 |  |
| *Pediococcus parvulus* | 2.11 | A | *Pediococcus parvulus strain S-182* | 99.93 | [PV300348](https://www.ncbi.nlm.nih.gov/nuccore/PV300348) |
|  |  |  | *Pediococcus inopinatus strain DSM 20285* | 98.56 |  |
|  |  |  | *Pediococcus damnosus strain DSM 20331* | 98.56 |  |
| *Pediococcus parvulus* | 2.11 | A | *Pediococcus parvulus strain S-182* | 99.79 | [PV300360](https://www.ncbi.nlm.nih.gov/nuccore/PV300360) |
|  |  |  | *Pediococcus damnosus strain DSM 20331* | 98.50 |  |
|  |  |  | *Pediococcus inopinatus strain DSM 20285* | 98.43 |  |
| *Pediococcus sp.* | 1.92 | B | *Pediococcus sp.* | 97.60 | [PV300336](https://www.ncbi.nlm.nih.gov/nuccore/PV300336) |
|  |  |  | *Pediococcus inopinatus strain DSM 20285* | 96.43 |  |
|  |  |  | *Pediococcus damnosus strain DSM 20331* | 96.43 |  |
| *Pediococcus parvulus* | 2.09 | A | *Latilactobacillus curvatus strain LMG 9198* | 99.57 | [PV300353](https://www.ncbi.nlm.nih.gov/nuccore/PV300353) |
|  |  |  | *Latilactobacillus graminis strain G90 (1)* | 99.18 |  |
|  |  |  | *Latilactobacillus sakei subsp. sakei strain DSM 20017* | 98.86 |  |
| *Pediococcus parvulus* | 2.06 | A | *Pediococcus sp.* | 98.63 | [PV300351](https://www.ncbi.nlm.nih.gov/nuccore/PV300351) |
|  |  |  | *Pediococcus inopinatus strain DSM 20285* | 97.46 |  |
|  |  |  | *Pediococcus damnosus strain DSM 20331* | 97.46 |  |
| *Weissella cibaria* | 2.02 | A | *Weissella cibaria strain II-I-59* | 99.73 | [PV300349](https://www.ncbi.nlm.nih.gov/nuccore/PV300349) |
|  |  |  | *Weissella confusa strain JCM 1093* | 99.18 |  |
|  |  |  | *Weissella muntiaci strain personal::8 H-2* | 97.54 |  |

Supplementary Table 2. The medium of pH value for the tested fermented products.

| Fermented products | The medium of pH |
| --- | --- |
| Ferm.BEET. | 3.70 ± 0.01 |
| Ferm.CARR. | 3.16 ± 0.19 |
| Ferm.CAUL. | 3.40 ± 0.24 |
| Ferm.CEL. | 3.05 ± 0.01 |
| Ferm.CUC. | 3.34 ± 0.01 |
| Ferm.CUC1. | 3.48 ± 0.01 |
| Ferm.CUC2. | 3.48 ± 0.01 |
| Ferm.KIM. | 3.40 ± 0.01 |
| Ferm.LEM. | 2.41 ± 0.01 |
| Ferm.RAD. | 3.28 ± 0.04 |

. Fermented materials: Ferm.BEET- fermented beetroot, Ferm.CARR.- fermented carrot, Ferm.CAUL.- fermented cauliflower, Ferm.CEL.- fermented celery, Ferm.CUC.- fermented cucumber, Ferm.CUC1.- fermented cucumber1, Ferm.CUC2.- fermented cucumber2, Ferm.KIM.- fermented kimchi, Ferm.LEM.- fermented lemon, Ferm.RAD.- fermented radish.
